# Targeting Beta-Blocker Drug–Drug Interactions with Fibrinogen Blood Plasma Protein: A Computational and Experimental Study

**DOI:** 10.3390/molecules25225425

**Published:** 2020-11-19

**Authors:** Michael González-Durruthy, Riccardo Concu, Laura F. Osmari Vendrame, Ivana Zanella, Juan M. Ruso, M. Natália D. S. Cordeiro

**Affiliations:** 1 LAQV-REQUIMTE, Department of Chemistry and Biochemistry, Faculty of Sciences, University of Porto, 4169-007 Porto, Portugal; riccardo.concu@fc.up.pt; 2 Soft Matter and Molecular Biophysics Group, Department of Applied Physics, University of Santiago de Compostela, 15782 Santiago de Compostela, Spain; juanm.ruso@usc.es; 3 Post-Graduate Program in Nanoscience, Franciscana University (UFN), Santa Maria 97010-032, RS, Brazil; laura.o.vendrame@gmail.com (L.F.O.V.); ivanazanella@gmail.com (I.Z.)

**Keywords:** drug–drug interactions, beta-blocker drugs, polypharmacology, molecular docking, DFT, ultrasound measurements

## Abstract

In this work, one of the most prevalent polypharmacology drug–drug interaction events that occurs between two widely used beta-blocker drugs—i.e., acebutolol and propranolol—with the most abundant blood plasma fibrinogen protein was evaluated. Towards that end, molecular docking and Density Functional Theory (DFT) calculations were used as complementary tools. A fibrinogen crystallographic validation for the three best ranked binding-sites shows 100% of conformationally favored residues with total absence of restricted flexibility. From those three sites, results on both the binding-site druggability and ligand transport analysis-based free energy trajectories pointed out the most preferred biophysical environment site for drug–drug interactions. Furthermore, the total affinity for the stabilization of the drug–drug complexes was mostly influenced by steric energy contributions, based mainly on multiple hydrophobic contacts with critical residues (THR22: P and SER50: Q) in such best-ranked site. Additionally, the DFT calculations revealed that the beta-blocker drug–drug complexes have a spontaneous thermodynamic stabilization following the same affinity order obtained in the docking simulations, without covalent-bond formation between both interacting beta-blockers in the best-ranked site. Lastly, experimental ultrasound density and velocity measurements were performed and allowed us to validate and corroborate the computational obtained results.

## 1. Introduction

Computational polypharmacology drug–drug interaction (DDI) studies are essential in rational drug-design and development [1,2,3,4,5,6,7]. Yet the exhaustive exploration of drug–drug side effects by experimental techniques remains a great challenge for the pharmaceutical industry owing to the involved costs and time. For example, beta-blocker DDI events constitute one of the most prevalent side effects associated with complications in several pharmaceutical therapies such as those employed to treat cardiovascular diseases. From the pharmacodynamic point of view, the DDI events refer to the simultaneous interactions of more than one drug (belonging to the same or different pharmacological groups) in the same molecular binding site of a given molecular target [2,8,9,10]. Polypharmacology DDI facets can affect millions of patients each year and apparently, the most feasible approach to address them is to use computational methods [4,5,6]. In fact, advanced computational methods such as machine learning, molecular dynamics, pharmacophore modeling, and molecular docking techniques have become powerful in silico tools at the initial stages of rational drug design [11,12,13,14,15,16,17]. Currently, the main limitation of these methods is to cope with the mechanistic interpretation of DDI phenomena at the atomic level [11,12,13,14]. Indeed, computational polypharmacology involves the characterization of DDI across multiple scales and organization levels by integrating drug specific binding properties [7]. Nevertheless, advanced molecular docking simulations combined with Density Functional Theory (DFT) calculations can be applied efficiently to model DDI events on the relevant binding sites of a given protein with atomic precision. Specifically in DDI studies, molecular docking simulations allow one to identify the stable drug–drug complexes by firstly predicting the feasible binding-sites of a target protein [13,17], followed by establishing from those, the best-ranked drug–drug binding configurations as well as their orientations within the best-ranked binding-sites along with the assessment of their binding affinity [11,18,19,20].

During the ligand-binding processes, it is common to find propensity of the ligands to interact with multiples binding sites within the same protein. The greater or lesser specificity of a given ligand to a particular binding site relies on the affinity at the biophysical environment where the interaction takes place, and the latter can be assessed by the druggability degree (D_g_) of the binding site [18,19,20]. D_g_ represents a quantitative estimation of the maximum intrinsic affinity of a given binding site of the protein to bind one or more drugs (ligands). Notice, however, that several pockets may have the ability to bind more than one drug enabling the occurrence of pharmacodynamics DDI, when partial or total overlapping of the drugs occurs at the same biophysical environment of a given binding site of the protein.

Despite the existence of great advances in computational pharmacology, how DDI phenomena can modulate the adverse effects at the molecular and atomistic level are still ignored. Previous experimental studies show that beta-blocker drug interactions can induce multiple adverse effects [21,22,23]. Particularly, when the beta-blockers acebutolol (A) and propranolol (P) are simultaneously administered in the bloodstream, they can induce different clotting perturbations due to interactions with proteins [24,25]. The most abundant protein circulating in the bloodstream plasma is fibrinogen, a protein with a molecular weight of ~340 kDa depending on A_α_ and B_β_ chains and the γ-γ and α-α crosslinking chains content [26]. From a structural and functional point of view, fibrinogen is a soluble glycoprotein with a central nodule (E-region) comprising several cavities and tunnels, which facilitate the ions, water solvent, and ligand transport, and in turn, its E-region has the maximum responsibility for the fibrin polymerization during the clotting process [27,28,29,30,31]. Therefore, the binding site localized in the most central part of the E-region of fibrinogen protein exhibits the greatest propensity for the occurrence of pharmacodynamic DDI leading to most of the side-effects among A and P beta-blockers.

In this work, we propose for the first time a combination of computational modeling approaches, based on molecular docking simulations and DFT calculations, along with experimental validation to study the drug–drug binding interactions between the beta-blockers A and P with the fibrinogen E-region (receptor).

## 2. Results and Discussion

One of the most important steps in computational rational drug-design is the precise prediction and characterization of the biological target structure [32]. A deep knowledge of its three-dimensional structure, flexibility properties, and residues composition of its catalytic binding site is crucial to understand the phenomena of ligand–protein interactions with relevance from the pharmacological point of view [33,34,35]. As referred before, during the ligand-binding processes in a given protein target, it is very common to find propensity of the ligand to interact with multiples catalytic binding sites (tiny cavities and tunnels) in the same protein [11,12,13,14,15,16,17]. Indeed, the greater or lesser specificity and selectivity of the ligand will be decided by the affinity at these biophysical environments where the interactions take place. Particularly, the DDI phenomena are crucial to prevent side effects and ensure the effectiveness and safety of drugs [1,2,3,4,5,6,7]. In this work, the study of polypharmacology DDI between A and P beta-blockers is tackled considering the most relevant fibrinogen E-region binding sites. Towards such purpose, we performed a pocket ranking of fibrinogen E-region binding sites by assessing their highest druggability D_g_ [36], as defined in the Material and Methods section (see Equation (8)). Notice that the higher D_g_ values are the maximum intrinsic affinity of the predicted binding sites of fibrinogen E-region with the ability to simultaneously bind the acebutolol and propranolol beta-blockers [36].

### 2.1. Prediction of the Binding Sites for Fibrinogen

**Figure 1:**
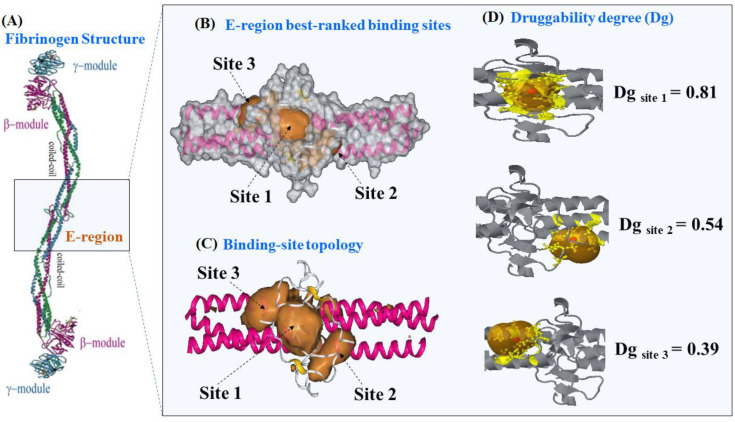
(**A**) Details on the molecular structure of fibrinogen protein with the relevant regions, such as the two C-terminal portions of the carbohydrate-linking coiled-coil-like chains (γ-module and β-module) and the funnel hydrophobic cavity thrombin binding-domain (E-region, PDB ID: 1JY2 with 1.4 Å of resolution), the latter highlighted with the light-blue rectangle. (**B**) 3D-DCNN prediction of the three most relevant fibrinogen E-region binding-pockets (site 1, site 2, and site 3) depicted as van der Waals surfaces. (**C**) Representation of the binding-site topology linked to catalytic active sites, depicted as volumetric orange regions. (**D**) Prediction of the corresponding D_g_ for the best three catalytic binding sites of the fibrinogen E-region, depicted as orange transparent hull surfaces. Therein, the alpha sphere centers for predictions are shown as small red points within each site and surrounding target residues (yellow colored).

Firstly, we present the predictions of the best-ranked catalytic binding sites of receptor fibrinogen obtained using the machine learning technique 3D-DCNN [32]. The 3D-DCNN algorithm performs a Delaunay triangulation with weighted points based on the computational geometric concept of the “alpha sphere” center [32,36,37]. An alpha sphere is a sphere which contains no internal atom and in turn, links four atoms on its border. In other words, the four atoms are at an equal distance to the alpha sphere center [32,36,37]. Then, the algorithm can detect plausible fibrinogen binding pockets by summing up all alpha sphere centers and later delimiting the access to cavities by scanning the van der Waals surfaces in the fibrinogen E-region. The procedure includes all the depth concave regions of fibrinogen that are directly associated with a high probability to ligand binding (higher D_g_ values) and exclude all the convex parts associated to low druggability [18,19,20]. The 3D-DCNN results showed that from a total of ten predicted binding sites in the fibrinogen E-region, the three best-ranked sites are the following ones depicted in Figure 1, namely: site 1 (D_g_ = 0.81) > site 2 (D_g_ = 0.54) > site 3 (D_g_ = 0.39).

**Figure 2:**
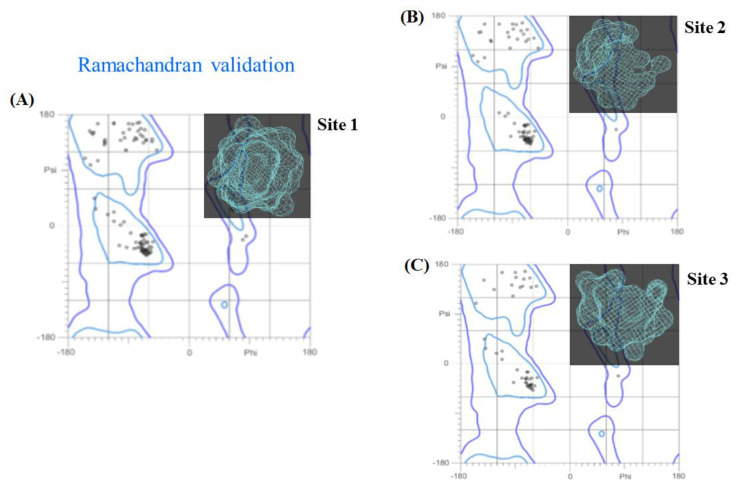
Ramachandran diagrams obtained for the best-ranked fibrinogen binding sites: (**A**) site 1, (**B**) site 2, and (**C**) site 3. For visualization purposes, these are also 3D-displayed in the upper right corner (light blue) of each panel. All the possible combinations of torsion dihedral angles Psi (ψ) vs. Phi (φ) are shown. Note the total absence of Ramachandran outlier residues for the three sites analyzed, usually located outside the Ramachandran colored purple contour, if any.

Let us now proceed to the 3D *X*-ray crystallographic structure validation of the fibrinogen binding sites. Figure 2 shows the Ramachandran plots for its three best-ranked binding sites. These plots contain a 2D-projection on the plane obtained from the 3D-crystallographic binding sites, pertaining to all their possible residues conformations according to the dihedral angles (Psi (ψ) vs. Phi (φ)) around the peptide bond of such residues. The allowed torsion values for the ψ vs. φ dihedral angles, placed within the Ramachandran colored purple contour, are identified as conformationally favored residues, otherwise, these are considered as sterically disallowed residues [38]. This validation has paramount importance in order to avoid the presence of potential false positives in the docking results coming from the target residues forming the E-region binding sites.

As can be seen in Figure 2, our results show that 100% of the residues belonging to the three validated binding sites can be categorized as conformationally favored residues with total absence of restricted flexibility. This fact clearly prevents the presence of potential false positives results in the modeling of the beta-blocker DDI systems, as well as ensuring the quality of our results from the conformational point of view. Additionally, it is important to note that in most docking studies, the Ramachandran validation is absent or in some cases inappropriately applied over all the protein. In this work, the Ramachandran X-ray crystallographic validation was performed for only the clusters of residues composing the relevant fibrinogen E-region binding sites (sites 1, 2, and 3), then allowing us to obtain a more adjusted evaluation of the conformational integrity of the target-residues potentially involved in DDI.

### 2.2. Identification of Tunnels for the Fibrinogen Binding Sites

Fibrinogen E-region binding-sites are highly complex systems containing a large and variety number of tunnels, clefts, grooves, protrusions, and empty spaces in the interior of the tangled funnel hydrophobic cavity formed by the γ-γ and α-α crosslinking from Aα and Bβ chains. These chains are located in the two outer sides of the binding sites for thrombin, which allow the supercoiling of protofibrils in the fibrinogen molecule during critical stages of the blood coagulation process [26,27,28,29]. Under the presence of ligands such as the A and P here studied, critical binding residues can be affected by modifications in the symmetry architecture of the Bβ-γ/Bβ-γ dimeric domain of E-region. In fact, it is well known that many β-adrenoreceptor blocking agents can affect the parameters of the blood coagulation such as fibrinogen concentration and activity when co-administered intravenously [24].

Let us now then focus our attention on the structural prediction and characterization of the tiny cavities (tunnels) composing the catalytic residues with the highest relevance-based druggability.

From the structural point of view, the tunnels are defined as entangled and small sub-cavities occupying the internal volume of the aforementioned binding sites, which selectively delimit the ligand transport to them, and usually present a narrower access determining the catalytic properties of the binding sites of the fibrinogen E-region. The specific functions of the tunnels include: (i) exchange of solutes, ions, and water between the tiny cavities; (ii) leading the transport of reaction intermediates between two distinct active sites; and (iii) maintaining the conditions of conformational and geometric complementarity for binding physiological substrates. Moreover, the residues forming the tunnel are considered as promising hotspots with high ligand-binding propensity [36]. Thus, taking into account the complexity of the tunnel hydrophobic cavity of the fibrinogen E-region [26,27,28,29], evaluating the catalytic function of fibrinogen residues on its detected tunnels is considered to be more informative than that of traditional predicted topological cavities.

**Figure 3:**
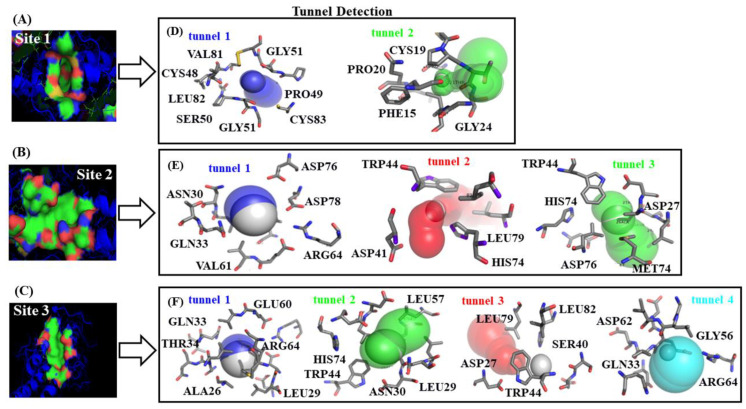
On the left, van der Waals surface representation of the three best-ranked fibrinogen binding-sites, namely: (**A**) site 1, (**B**) site 2, and (**C**) site 3. Therein, red and blue regions represent acid and basic residues, respectively. On the right, (**D**–**F**) representation of the different tiny cavities such as tunnels detected for each predicted fibrinogen binding site with the corresponding surrounding target-residues.

According to this and by resorting to Caver software [36], we identified at least two tunnels geometrically interconnected for each predicted catalytic binding-site (sites 1–3), sharing catalytic binding residues (see Figure 3).

It is important to highlight here that binding sites with high druggability comprise target-residues displaying higher tolerance for deep environments such as: CYS, ALA, ILE, LEU, MET, PHE, VAL, and TRP. The latter in turn directly influence the ligand binding properties of the established protein–ligand complexes (A + P/fibrinogen)—i.e., their specificity, enantioselectivity, inhibition constants, and thermodynamic stability [39,40]. As mentioned before, we also identified the “worst fibrinogen binding site” to be used as reference control denoting the absence of beta-blocker drug–drug interactions. For this instance, a small region formed by two different tunnels located in opposite crystallographic planes of the fibrinogen E-region was identified and showed to have the lowest value of druggability degree (D_g_ = 0.08). More details concerning the worst fibrinogen binding site can be found in Figure S1 of the Supplementary Material.

### 2.3. Calculation of Energetic Contributions for Binding Affinity

Moving on to the docking experiments, now we will focus our attention on the study of potential DDI phenomena between A and P beta-blockers with the receptor fibrinogen E-region. Since several tunnels from the three binding sites were detected in the fibrinogen E-region (Figure 3), we established three pocket ranking prioritization criteria. The first one is the mutual overlap criterion (MOc) [37], which considers a given binding site likely relevant for DDI, if partial or total ligand-overlapping exists at the same crystallographic plane or biophysical environment in that site. Additionally, it considers the site that is likely relevant, if at least > 50% of the ligand atoms are within 3 Å of at least one alpha sphere. The second criterion pertains to the ligand-docking complexes with spontaneous thermodynamic process (Δ*G*_bind_ < 0 kcal/mol). With regard to this criterion, as already referred to, the docking simulation results are categorized as energetically unfavorable when the Gibbs free energy of the formed complexes is Δ*G*_bind_ ≥ 0 kcal/mol, pointing either to extremely low or complete absence of binding affinity, otherwise they are categorized as having medium to high docking affinity [41,42]. Finally, the third criterion relates to the high depth of the target-residues of relevant binding sites. The depth is defined as the distance of any atom in a given residue to its closest water molecule from virtual bulk solvent [39,40]. In this instance, it is important to note that the cited crystallographic water molecules were removed from the binding sites before starting the molecular docking simulations during the step of receptor preparation (see Materials and Methods section).

**Figure 4:**
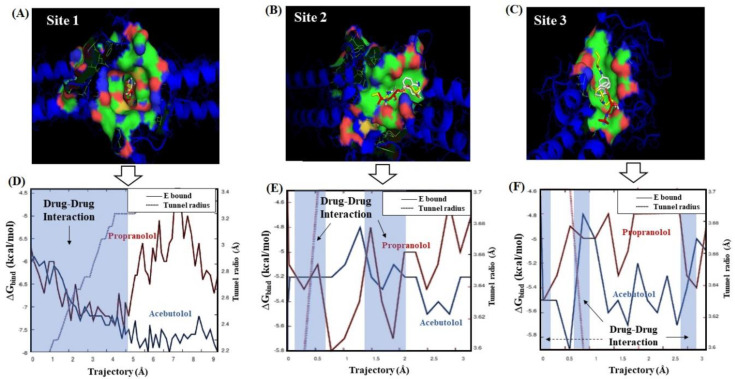
On the top, van der Walls surface representation for the best drug–drug interaction (DDI) systems forming stable docking complexes with the fibrinogen E-region binding sites. (**A**) A plus P at site 1; (**B**) A plus P at site 2; and (**C**) A plus P at site 3. On the bottom, panels (**D**–**F**) show the individual Gibbs free energy profiles (Δ*G*_bind_) from both beta-blockers plotted as a function of the trajectory and the fibrinogen tunnel radius across the predicted catalytic sites. Therein, the corresponding energy profile of A and P is depicted in red and blue solid lines, respectively. The transparent blue rectangle with the black arrow in each panel indicates the occurrence of DDI events—i.e., A and P interacting at the same biophysical environment in sites 1, 2, and 3. For comparison purposes, please refer to Figure S2, where the LTA results for the worst fibrinogen binding site are shown.

Several factors can influence computational polypharmacology DDI studies. Amongst others are the protein’s crystallographic quality, its flexibility properties, the pharmacological relevance of the catalytic residues involved in the polypharmacology interactions, the physico-chemical properties of the interacting ligands, and the presence of different conformations within the binding sites. Particularly, the thermodynamic factors are crucial to tackle the polypharmacology DDI mechanisms. To address the latter, we carried out a ligand transport analysis for the obtained DDI complexes between the beta-blockers A and P with the three best-ranked binding-sites. The LTA-approach executes an iterative docking algorithm which simultaneously scans the binding-energy trajectory vs. tunnel radius under spatially restrained conditions, considering the same biophysical environment for both beta-blockers to every slice of the binding sites (see Figure 4).

In general terms, the obtained beta-blocker binding energy trajectories fit with a spontaneous thermodynamic process (Δ*G*_bind_ < 0 kcal/mol), therefore suggesting the formation of stable beta-blocker drug–drug complexes in the three predicted fibrinogen binding sites. According to this, the modeling results on the throughput tunnel parameter did not show significant differences between the three best-ranked binding sites. In fact, the estimated ***T*** values from the predicted tunnels were high and very close to each other (i.e., ***T***_[site 1-tunnel 2]_ = 0.90, ***T***_[site 2-tunnel 1]_ = 0.95, and ***T***_[site 3-tunnel 1]_ = 0.96), indicating that the three binding sites display a similar performance for the transport of both beta-blockers. Concerning this, note that ***T*** values close to 1 reflect a high probability of these tunnels for the simultaneous transport of beta-blocker ligands increasing the probability of DDI. However, considering the obtained Δ*G*_bind_ values from the LTA-analysis, one can clearly distinguish that the DDI phenomenon hierarchically follows the order: site 1 > site 2 > site 3. In the case of the trajectories through the binding site 1, the obtained binding energy profiles for both beta-blockers suggest the formation of a DDI complex with a high stability with a range of Δ*G*_bind_ energy starting from −5.8 to −7.40 kcal/mol. Additionally, the interaction affinity soared significantly in the binding site 1 around the tunnel radius (≈2.83 Å) until its critical value (≈2.4 Å) for both beta-blockers, which fits with the maximum of drug–drug coupling interaction near to the narrowest part of the binding site 1. After this, the drug–drug trajectories became abruptly independent for both beta-blockers. Interestingly, beta-blocker A continued interacting at site 1 and increased its binding affinity from −7.40 kcal/mol until a maximum value of Δ*G*_bind_ = −7.70 kcal/mol. In contrast, P showed several peaks of significant loss of affinity in the energy range of −7.40 and −6.5 kcal/mol in that site. According to the binding trajectory of P, several factors are likely to explain its behavior, such as rotational and repulsive forces linked to different binding modes, as well as clashes of P with the residues surrounding tunnels 1 and 2 of site 1. In fact, these likely induce a relative loss of the P affinity under the influence of A at the same biophysical environment. Recent experimental evidence using small-angle X-ray scattering (SAXS) also suggests that P affects the fibrinogen protein in a more significative way than A. However, we firmly believe that A induces a reversible loss of P affinity [43], which can be verified in the range of 7–9 Å for the isolated trajectory of P (see Figure 4D).

Instead, the obtained trajectories for A and P in sites 2 and 3 exhibited a non-continuous DDI profile (i.e., regions where the binding trajectories are independent or non-associated) with an average affinity around −5.22 kcal/mol. In both these sites, a similar behavior happened in the first steps of the beta-blocker transport around tunnel radius ≈3.5 Å. In addition, one can detect the absence of DDI trajectories between 0.5–1.4 Å and 0.2–0.5 Å for sites 2 and 3, respectively. In site 2, the absence of DDI trajectories may be associated with the reduction in the A affinity (Δ*G*_bind_ ranges from −5.2 to −4.9 kcal/mol) and, in site 3, with the reduction in P affinity (Δ*G*_bind_ ranges from −5.5 to −4.9 kcal/mol). So, this suggests that these differences between the beta-blocker trajectories could be attributed to their transport through different tunnels, and the depth of the surrounding target-residues composing the tunnels of both sites. From the 2 Å trajectory for both beta-blockers in the binding sites 2 and 3, one can see the absence of DDI in a large part of the transport of these beta-blockers associated to a loss of P affinity, may be due to the presence of A interacting simultaneously with the same target-residues (vide infra), clearly affecting the thermodynamic stabilization of P interactions in such sites. It is important to note that in the binding sites 2 and 3, the apparent similarity of the beta-blocker drug–drug trajectories could be attributed to a quasi-symmetrical architecture of these sites with respect to the main center of tunnel-hydrophobic cavity (site 1) in the fibrinogen E-region [26]. In addition, we checked the mutual overlap criterion by performing a control of simulation experiment based on the LTA approach for the worst fibrinogen binding site (D_g_ = 0.08) [36,37]. In doing so, it was corroborated that the beta-blocker trajectories for A and P do not intercept during the passage through this site (see Figure S2). This result is of great theoretical relevance because it serves as a reference to identify the total absence of DDI patterns when compared to the interactions in sites 1, 2, and 3.

**Figure 5:**
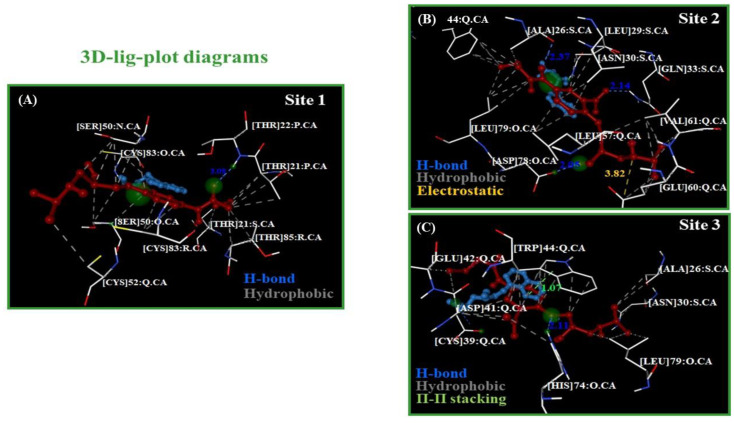
Representation of 3D-lig-plots diagrams for the best beta-blocker drug–drug binding-poses. (**A**) A plus P at site 1; (**B**) A plus P at site 2; and (**C**) A plus P at site 3 (A displayed in red color and P in blue). Therein, the atoms from target-residues colored in green correspond to potential hotspots for maximum drug–drug interactions between both beta-blockers in each fibrinogen binding site evaluated.

Following this idea, to verify the presence or absence of DDI for beta-blockers A and P at the same biophysical environment in the best-ranked fibrinogen binding sites, the corresponding 3D-ligand plot diagrams [44,45] were determined and are shown in Figure 5.

As can be seen, the A molecule involves a greater number of target-residues compared to P, covering a greater area in the biophysical environment shared by both beta-blockers during the docking interactions in the three binding sites. In all the cases, the best drug–drug binding pose (A plus P) showed an extended shape to interact with the three fibrinogen sites. Additionally, considering the approximate lengths of both molecules (A: ≈16.1 Å, and P: ≈4.8 Å), this suggests that the pharmacodynamic DDIs are mostly influenced by the contribution of the atoms from beta-blocker A, since it displayed a greater number of hydrophobic contacts with binding residues following the “mutual overlap criterion” cited above.

An overview of the results revealed that the most relevant beta-blocker DDIs are the hydrophobic (N···C···C)-backbone side chain non-covalent interactions [46,47]. A greater number of these pertain to binding site 1 when compared to sites 2 and 3, which show a similar proportion. Thus, the presence of multiples hydrophobic (N···C···C)-backbone side chain non-covalent interactions between the beta-blockers (A > P) with the fibrinogen binding sites promote thermodynamic destabilizing effects on the residue site-chain packing [46,47], inducing local perturbations in the flexibility and ligand-binding properties of those binding sites (site 1 > site 2 > site 3). Furthermore, other detected relevant interactions for these beta-blockers are an electrostatic (attractive interaction) between the electron-acceptor (N^+^)-(A) atom with two (O^−^)···δ-GLU60: chain Q (site 2) and a strong hydrophobic π···π stacking interaction between the naphthalene moiety belonging to P with the aromatic heterocyclic N-indole moiety-TRP44: chain Q (site 3), which can influence the stabilization of the A molecule that shares the same binding residue (TRP44: chain Q) through hydrophobic interactions with the benzene ring that is part of that aromatic heterocyclic *N*-indole moiety.

Another aspect deserving special attention for targeting the beta-blocker DDI is the formation of a stable multicomponent docking complex that follows a recognized heterogeneous Monod-Wyman-Changeux Allosteric Transition model (HMWC-AT) [48,49,50], which depends on the fractional occupancy parameter (*δ_site_*) as a measure of the affinity constant (*K_i_*) or degree of saturation from the different binding sites [48,49,50]. The HMWC-AT model is related to the cases where two or more ligands interact with different binding sites of a given target-protein, but where just one affinity (∆*G*_bind_) per ligand is attributable [49]. In this context, the fractional occupancy for a given fibrinogen binding site forming a multicomponent complex with two different ligands (A and P) can be defined per binding site as follows:

(1) $$\begin{array}{ll} \delta_{\mathrm{site}} & =\frac{1}{n}\;\{\frac{\left[\sum_{i=1}^{n_{1}}(|A|K_{i}^{R}\;\prod_{j\neq i}(1+|A|K_{j}^{R}))\right]\times \left[\sum_{i=1}^{n_{2}}(|P|K_{i}^{R}\;\prod_{j\neq i}(1+|P|K_{j}^{R}))\right]}{\prod_{i=1}^{n_{1}}(1+|X_{1}|K_{i}^{R})\;\prod_{i=1}^{n_{2}}(1+|X_{2}|K_{i}^{R})+L\;\prod_{i=1}^{n_{2}}(1+c_{i}|X_{1}|K_{i}^{R})\;\prod_{i}^{n_{2}}\left(1+c_{i}|X_{2}|K_{i}^{R}\right)}\\ & +\frac{L\;\left[\sum_{i=1}^{n_{1}}(c_{i}|A|K_{i}^{R}\;\prod_{j\neq i}(1+c_{j}|A|K_{j}^{R}))\right]\times \left[\sum_{i=1}^{n_{2}}(c_{i}|P|K_{i}^{R}\;\prod_{j\neq i}(1+c_{j}|P|K_{j}^{R}))\right]}{\prod_{i=1}^{n_{1}}(1+|A|K_{i}^{R})\;\prod_{i=1}^{n_{2}}(1+|P|K_{i}^{R})+L\;\prod_{i=1}^{n_{2}}(1+c_{i}|A|K_{i}^{R})\;\prod_{i=1}^{n_{2}}\left(1+c_{i}|P|K_{i}^{R}\right)}\} \end{array}$$

In this equation, the first ligand (Acebutolol: A) interacts with the *n*_i_ fibrinogen binding sites (*n*_1_: sites 1, 2, or 3), and the second ligand (Propranolol: P) simultaneously interacts with the same *n_i_* fibrinogen binding sites (*n*_2_: sites 1, 2, or 3), but with different affinity (*K_i_* or *K_j_*) and both with the best D_g_. Furthermore, *X*_1_ and *X*_2_ denote the ligand concentration of the evaluated beta-blockers, and the parameter *L* is an isomerization constant of the fibrinogen E-region formed by A_α_ and B_β_ chains with pseudo-allosteric behavior. This parameter is obtained by the ratio: *L* = |*T*_0_|/|*L*_0_|, which describes the equilibrium between the high-affinity relaxed conformation state (*R*_0_) and the low-affinity tensor conformation state (*T*_0_) for the three best-ranked fibrinogen binding sites [48,49,50,51]. In this context, the different affinity (*K_i_*) is represented in the high-affinity *R*-state like $K_{i}^{R}$ for both beta-blockers, and the *c* parameter indicates how much the equilibrium between *T* and *R* states changes under beta-blocker interactions [50,51,52]. The value of D_g_ ≈ Δ*G*_max_ (Fibrogen site) is a function directly associated to the fractional occupancy parameter (*δ*_site_), namely: *δ*_site 1_ ≈ 0.81 > *δ*_site 2_ ≈ 0.54 > *δ*_site 3_ ≈ 0.39.

**Figure 6:**
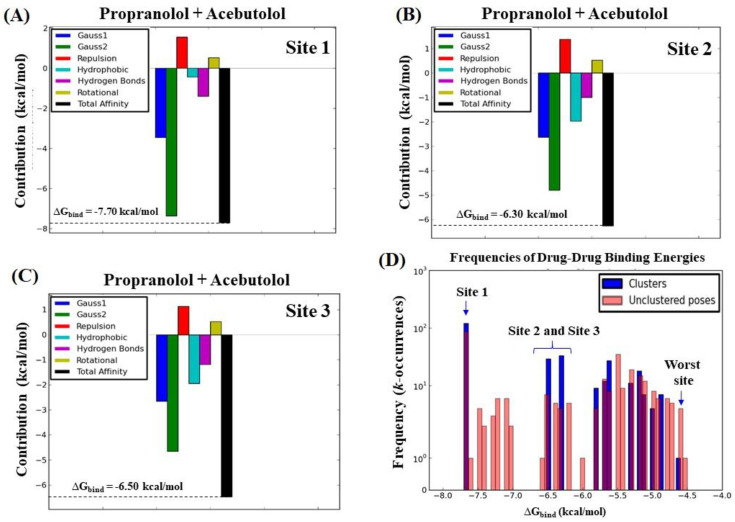
Graphical breakdown of the different binding energy contributions of the total binding affinity ($\Delta G_{\mathrm{bind}}^{T}$) with the corresponding values obtained from the simultaneous interaction of both beta-blockers with the fibrinogen E-region binding sites. (**A**) A and P at site 1 ($\Delta G_{\mathrm{bind}}^{T}$ = −7.70 kcal/mol); (**B**) A and P at site 2 ($\Delta G_{\mathrm{bind}}^{T}$ = −6.30 kcal/mol); and (**C**) A and P at site 3 ($\Delta G_{\mathrm{bind}}^{T}$ = −6.50 kcal/mol). Therein, different colors were used to distinguish the type of thermodynamic contribution, namely: attractive energy dispersion based on two Gaussian functions (Gauss1 and Gauss2 in blue and green, respectively), repulsion energy (red), hydrophobic energy (light blue), hydrogen bond energy (purple), rotational energy (yellow), and total affinity (black). (**D**) Frequency distribution of the total binding energies, in which the *x*-axis pertains to the drug–drug total affinities ($\Delta G_{\mathrm{bind}}^{T}$) and the *y*-axis to their frequencies of occurrence (*k*-occurrences), obtained from both the docked drug–drug poses (blue bars) and the undocked drug–drug poses (pink bars).

Let us now begin by ascertaining the total binding affinity ($\Delta G_{\mathrm{bind}}^{T}$), as well as its thermodynamic contributions (∆*G*_Gauss1_, ∆*G*_Gauss2_, ∆*G*_repulsion_, ∆*G*_H-bond_, ∆*G*_hydrophobic_, and ∆G_rot_; see Equations (11) and (12) in the Materials and Methods section). Additionally, the frequencies (*k*-occurrences) of affinities were evaluated by assuming that the ligand binding occurs in the high-affinity relaxed conformation state (*R*_0_) in the three best-ranked binding sites (site 1 > site 2 > site 3). The results of this analysis are shown in Figure 6.

An analysis of the results depicted in Figure 6 revealed that the most dominant energy contributions for the total binding affinity $\Delta G_{\mathrm{bind}}^{T}$ of the beta-blocker DDI systems are the pair of steric Gaussian molecular mechanism distance-dependent terms (Gauss2 > Gauss1) [53]. In particular, the Gauss2 energetic terms provide relevant information about the influence of attractive binding energies of the beta-blockers system with the three fibrinogen binding sites (site 1 > site 2 ≈ site 3). These show the presence of the strongest interactions based on non-covalent hydrophobic and non-electrostatic attractive energy contributions between the A and P hydrophobic atoms making the DDI system. This indicates that the great influence exerted by the Gauss2 energy [53] is due to the formation of temporary dipoles (fluctuating dipole–induced dipole) with electron cloud overlap between both beta-blockers. It is well-known that this phenomenon can induce random fluctuations of electron density and non-zero instantaneous dipole moments in all the interacting atoms, therefore leading to a significantly increase in the total affinity when both beta-blockers interact at the same biophysical environment in the fibrinogen E-region binding site. Particularly, based on the Gauss2 attractive energy contributions, the narrowest pocket seen in the case of the binding site 1 appeared to favor a steeper stabilization of the drug–drug system when compared to binding sites 2 and 3. Indeed, the later sites presented a greater number of tunnels and binding interaction surfaces that decrease the probability of drug–drug interactions based on their frequency (*k*-occurrences). These conclusions match up with the previous results obtained by the ligand transport approach [54], where very close values of maximum affinity were found for sites 2 and 3 compared to the first-ranked binding site 1. In addition, the hydrogen-bond interactions contribute to the stabilization of the beta-blocker drug–drug complexes in the system but show less impact on its total affinity, which display similar interaction values for the three catalytic active sites studied. On the other hand, from a quantitative point of view, the energetic contributions related to repulsive and rotational forces showed a similar behavior for the three binding sites. So, repulsive and rotational forces are thought to have low relevance for the stabilization of the beta-blocker drug–drug complexes in the fibrinogen E-region binding sites evaluated. Furthermore, the energetic contributions of the beta-blockers A and P with the “worst fibrinogen binding site” were used as a control simulation experiment for comparison purposes (see Figure S3).

On the other hand, the results shown in Figure 6D corroborate that the beta-blocker DDI events present a higher prevalence in the fibrinogen E-region site 1 compared to sites 2 and 3, based on counting the binding affinity frequencies (*k*-occurrences) [54]. In fact, the calculated values of *k*-occurrences in site 1 (*k* = 100) were significantly higher compared to those in sites 2 and 3 (*k* = 10–13), suggesting the high propensity of the fibrinogen E-region binding site 1 for DDI events [53]

**Figure 7:**
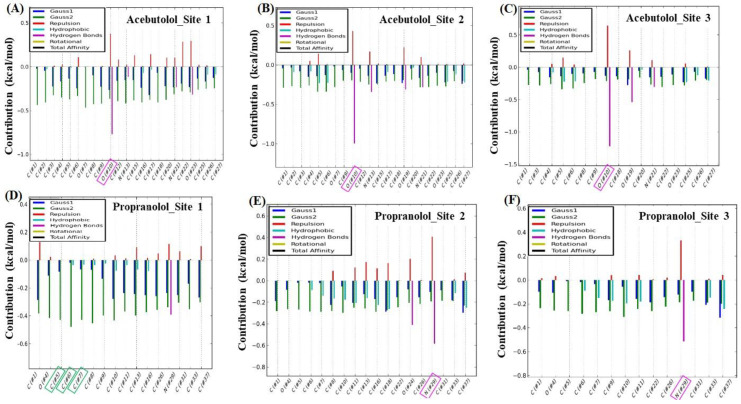
Graphical representation based on the beta-blocker per atom energy contributions to the individual binding affinity ($\Delta G_{\mathrm{bind}}^{A}$ or $\Delta G_{\mathrm{bind}}^{P}$) in the three best-ranked binding sites. On the top, energy contributions for each atom of A interacting with the three best-ranked binding sites: (**A**) A-oxygen-(2)-atom/site 1, (**B**) A-oxygen-(2)-atom/site 2, and (**C**) A-oxygen-(1)-atom/site3. On the bottom, energy contributions for each atom of P interacting with the three best-ranked binding sites: (**D**) P-C-atoms (C2, C3, C4)/site 1, (**E**) P-N-atom/site 2, and (**F**) P-N-atom/site 3. Please note that therein the symbol (#) *x-*axis is only used for labeling purposes and does not fit with the atomic position in the beta-blocker molecular structures.

To complement the previous analysis, we carried out additional control simulation experiments in order to identify which specific atom, or set of atoms, of the beta-blockers could present the maximum energy contribution to the individual binding affinities ($\Delta G_{\mathrm{bind}}^{A}$ or $\Delta G_{\mathrm{bind}}^{P}$), taking into account again the same thermodynamic energy contributions. To this end, a per atom energy contribution analysis was performed separately for each beta-blocker (see Figure 7). In fact, such analysis allows a more detailed evaluation of the contribution of the individual atoms of the beta-blockers interacting separately with the fibrinogen E-region binding sites [33,34,41,42,55,56].

As can be observed, for molecule A, the largest energetic contribution was given by the H-bond interaction generated by the A (H)···O-atom-(#2), with an oxo-group with electron-acceptor properties that interacted with the (H)-THR22: chain P:CA in binding site 1 (Figure 7A). In site 2, the highest energetic contribution for molecule A was supplied by means of H-bond interaction between the (H)-axial-(#2) of the (OH)-hydroxyl group of A, which exhibited electron-donor properties to interact with the (H)···ASP78: chain O: CA (Figure 7B). In the case of site 3, the atomic energy contribution was provided by the H-bond interaction between the (H)···O-atom-(#1)-A, which interacted with the (H)-axial-(#2) of the (OH)-HIS74 hydroxyl group in a similar way to the previous interaction in chain O (Figure 7C). The maximum per atom energetic contributions for the A molecule to $\Delta G_{\mathrm{bind}}^{A}$ were then provided by hydrogen bond interactions, these being remarkably close in the three fibrinogen E-region binding sites (Δ*G*_H-bond_ ≈ −0.8 kcal/mol).

Regarding the P molecule, the maximum per atom energy contribution to the individual $\Delta G_{\mathrm{bind}}^{P}$ was provided by a triad of C-atoms: C-atom (#2) > C-atom-(#1) ≈ C-atom-(#3) in order of influence, with the SER50:chain Q:CA interacting through the hydrophobic (N···C···C)-backbone and site chain (based on the attractive Gauss2 energy of ca. −0.5 kcal/mol) in site 1 (Figure 7D), and also inducing hydrophobic clashes. In binding site 2, the most relevant atomic energy contribution of the P molecule was provided by means of Δ*G*_H-bond_ interaction between the electron-acceptor (H)···N-acylated-atom-(#1) of P, which interacted with the (H)···N-atom of the ASN30: chain S:CA (Figure 7E). Lastly, in site 3, the most significant energetic contribution to $\Delta G_{\mathrm{bind}}^{P}$ was led by Δ*G*_H-bond_ involving the same electron-acceptor (H)···N-acylated-atom-(#1) in the P molecule, which interacted with the (H)-atom linked to the (H)···O-CYS39: chain Q: CA (Figure 7F). These two lasts per atom energetic contributions of the P molecule interacting with sites 2 and 3 exhibited remarkably close values of Δ*G*_H-bond_ (≈−0.5 kcal/mol). Additional details such as control simulation experiments for the “worst fibrinogen binding site” are provided in Figure S4 of the Supplementary Material.

**Figure 8:**
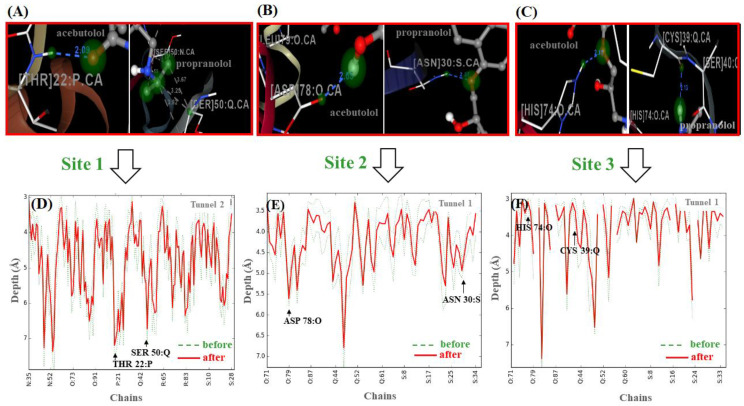
On the top, for each beta-blocker, critical target-residues interacting with the ligand atoms that showed the maximum atom energy contribution to the individual binding affinity. (**A**) THR22:P_site1/(A)-oxygen-(2)-atom and SER50_site1: Q/(P)-C-atoms_ (C2, C3, C4); (**B**) ASP78:O_site2/(A)-oxygen-(2)-atom and ASN30:S/(P)-N-atom; (**C**) HIS74:O_site3/(A)-oxygen-(1)-atom and CYS39: Q_site3/(P)-N-atom. On the bottom, (**D**–**F**) correspond to the depth profiles (*D*_[*c*,*i*]_ vs. chains) for the critical target-residues linked to their corresponding side-chains into the relevant tunnels. Therein, the amplitude of the depth peaks was evaluated before (green dashed lines) and after beta-blocker interactions (red solid lines).

Let us now assess the perturbations in the depth profiles of target residues making relevant tunnels (THR22:P, SER50: Q/site1, ASP78:O, ASN 30:S/site2 and HIS 74:O, CYS39: Q/site3) that interacted with the beta-blocker ligand atoms previously identified, with the maximum per atom energy contribution to the individual binding affinities [39,40,44] (see Figure 8).

According to the results shown in Figure 8 (panels: D–F), the relationship between the pharmacodynamic drug–drug interactions and the perturbations on the depth values for the revealed target-residues of A-A and P-P could be modeled by incorporating the latter into Equation (12) shown in the Material and Methods section, leading to expressions for $D_{[\mathrm{THR}22\cdots \mathrm{SER}50]}$, $D_{[\mathrm{ASP}78\cdots \mathrm{ASN}30]}$, and $D_{[\mathrm{HIS}74\cdots \mathrm{CYS}39]}$. In those expressions, it appears the $q_{i}^{c}$ descriptor (i.e., $q_{\mathrm{THR}22}^{P}$, $q_{\mathrm{SER}50}^{Q}$, $q_{\mathrm{ASP}78}^{O}$, $q_{\mathrm{ASN}30}^{S}$, $q_{\mathrm{HIS}74}^{O}$, and $q_{\mathrm{CYS}39}^{Q}$), derived from the distance matrix between the 20 standard amino acids, which depends on the frequency of the target residues in a certain position for a given chain (*c*). As referred to in that section, this descriptor might be used as an adjusted residue-binding probability to establish the quasi-sequence-position-order descriptor $J_{i}$ [39,40,44], which depends of the position (*i* and *i + d*) and the frequency-based occurrence of the target residues in the chain sequence. In this context, for each target residue type, the depth descriptor $q_{i}^{c}$ is defined by means of the following Equation:

(2) $$q_{i\;(J_{i})}^{c}=\frac{f_{r}}{\sum_{i=1}^{20}f_{r}+w\sum_{d=1}^{L}\tau_{d}}\;|\;i_{\text{-}\mathrm{target}}=1,\;2,\ldots ,\;20$$

in which $\tau_{d}$ is defined as follows:

(3) $$\tau_{d}=\sum_{i=1}^{N-d}{\left(d_{i,[i+d]}\right)}^{2}\;|\;d=1,\;2,\ldots ,\;L_{\max}$$

**Figure 9:**
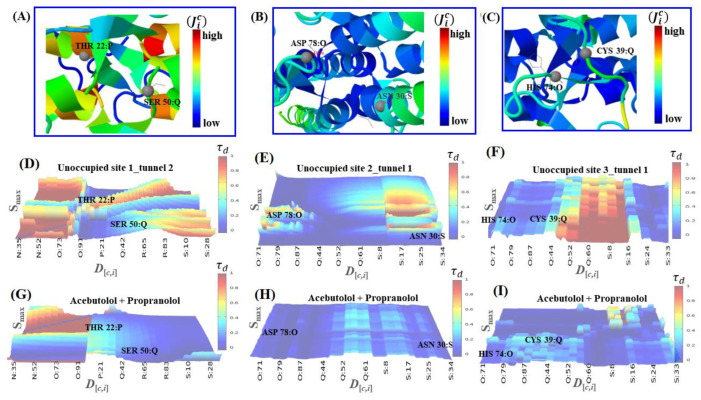
On the top, graphical representation of the beta-blocker binding ability for critical fibrinogen target-residues based on the depth quasi-sequence-position-order descriptor (*J_i_*). (**A**) THR22:P, SER50: Q; (**B**) ASP78:O, ASN30:S; and (**C**) HIS74:O, CYS39:Q. Orange and red colored regions depict high binding ability for the beta-blockers, while blue ones show low binding ability. On the bottom, 3D-surface maps of the quasi-sequence-order-coupling descriptor ($\tau_{d}$) as a function of residue depth *D*_[*c*,*i*]_ vs. maximum solvent accessibility. The simulation conditions considered to study the depth perturbation on the aforementioned target-residues are: (**D**) unoccupied site 1 (tunnel 2); (**E**) unoccupied site 2 (tunnel 1); and (**F**) unoccupied site 3 (tunnel 1). (**G**–**I**) The later panels refer to the last simulation condition considered—that is, the study of the residue depth perturbations of the aforementioned target-residues under the influence of drug–drug interactions with both beta-blockers A plus P. Therein, the color bar on the right of each map represents the residue depth perturbations based on the values of descriptor $\tau_{d}$, which range from weak (blue) to strong (orange/red) ones.

In Equation (2), $f_{r}$ stands for the normalized frequency-based occurrence of the target residue analyzed (i.e., THR22: P, SER50: Q/site 1, ASP78:O, ASN30:S/site 2 and HIS74:O, CYS39: Q/site 3), and *w* is a weighting factor (*w* = 0.1). In Equation (3), the parameter $\tau_{d}$ stands for the *d*-th rank sequence-order-coupling descriptor based on the C-(α)-distances (*d_i_*_,[*i*+*d*]_) between the two neighbors most contiguous residues around the target-residue at the position (*i*, *i* + *d*), and *L*_max_ for the maximum length of the chain (*c*), where the fibrinogen target-residues are included. Then, one can estimate the residue depth-perturbations after the beta-blocker interactions ($J_{c}^{i}$) by correcting this descriptor with an adjusted residue-binding probability under the influence or binding perturbation with the beta-blocker ligands (*lig_β_*_-*blocker*_: A and P)—i.e., site 1: $J_{\mathrm{THR}22/A}^{P}$, $J_{\mathrm{SER}50/P}^{Q}$; site 2: $J_{\mathrm{ASP}78/A}^{O}$, $J_{\mathrm{ASN}30/P}^{S}$; and site 3: $J_{\mathrm{HIS}74/A}^{O}$, $J_{\mathrm{CYS}39/P}^{Q}$. From the results shown in Figure 8 (panels D, E, and F), it can be seen that, in general terms, the beta-blocker drug–drug interactions induced a decrease in the residue depth values (*D*_[*c*,*i*]_) of approximately 1–2 Å for the target-residues analyzed. Such results, therefore, suggest that the simultaneous interactions of both beta-blockers can induce loss of coupling between the target-residues (i.e., changes in the parameter $\tau_{d}$), leading to anisotropic changes in the C-(α)-distances of the interacting target residues from their equilibrium coordinates, and depth-perturbations (i.e., displacements) measured by the *D*_[*c*,*i*]_ parameter. Additionally, these molecular changes could significantly affect the substrate-specificity for thrombin—the natural ligand substrate of the fibrinogen E-region, and flexibility properties of the fibrinogen binding sites [39,40,44] (see Figure 9).

As can be seen, the fibrinogen site 1 led preferably to beta-blocker DDIs considering that the modifications in the residue depth profiles (THR22:P, SER50: Q) were more pronounced compared to those on site 2 (ASP78:O, ASN30:S) and site 3 (HIS 74:O, CYS 39: Q) after these occurred. Furthermore, considering the different fibrinogen target-chains *c*, one can see that tunnel 2 at fibrinogen site 1 was the most preferred biophysical environment for the beta-blocker drug–drug interactions. Indeed, tunnel 2 at site 1 showed the highest relevance involving all the fibrinogen target-chains (N, O, P, Q, R, S) with *c*_[site 1]_ = 6 compared with that at tunnel 1 of sites 2 and 3 that display medium relevance (*c*_[site 2]_ = *c*_[site 3]_ = 3) and involving only the chains O, Q, and S. Additionally, the occurrence of the beta-blocker DDI phenomena was more likely at site 1 than sites 2 and 3 since it obeys a tighter relationship with D_g_, S_max_, and *D*_[*c*,*i*]_ (see Figure S5).

From the polypharmacology point of view, these results have a remarkable value since they match with previous in vitro evidence, which suggest that modifications in the residue depth profile can significantly affect several binding properties of the catalytic sites such as substrate-specificity, enantioselectivity, inhibition constants (*K_i_*), and thermodynamic-stability of the protein–ligand complexes [39,40].

### 2.4. DFT Modeling of Beta-Blocker Drug–Drug Binding Systems

**Figure 10:**
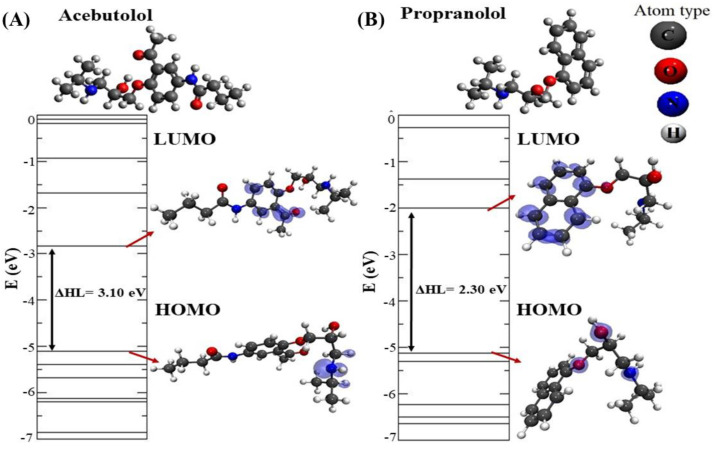
Representation of the electronic energy levels and local charge density plots with the corresponding highest occupied molecular orbital (HOMO) and lowest unoccupied molecular orbital (LUMO) levels (4.3 × 10^−3^ e/Å^3^ iso-surface) for the optimized beta-blocker ligands obtained by Density Functional Theory (DFT) calculations. (**A**) Beta-blocker A and (**B**) beta-blocker P. In the lower right corner, the atom types of the beta-blockers are depicted, namely: C-atoms (dark gray), O-atoms (red), N-atoms (blue), and H-atoms (light gray).

In order to further characterize the structural and electronic properties of the most stable beta-blocker drug–drug binding configurations, a DFT study was then carried out [57]. We started by evaluating the structural and electronic properties of the isolated beta-blockers A and P (see Figure 10).

To do so, we calculated the electronic levels of the isolated beta-blockers with the corresponding local charge densities plots for the highest occupied molecular orbital (HOMO) and the lowest unoccupied molecular orbital (LUMO) [58,59]. These molecular orbitals ascertain the way the molecule interacts with other species and allow one to predict the nature of its reactivity, physical, and structural properties. In particular, the HOMO-LUMO energy gap can be used as a metric of the stability of interactions established by such beta-blockers [60].

Here, it should be noted that local charge density plots allow us to identify relevant target-atoms potentially involved in the beta-blocker DDI from an electronic point of view [58,61]. For molecule A, the obtained HOMO–LUMO energy gap difference (∆HL) was 3.10 eV, and, as can be seen, the density of charges in the HOMO level was essentially placed in the N-atom, while in the LUMO level, those were more concentrated in the (*sp*^2^)-C-atoms of the aromatic ring, including the O-atom from one part of this molecule. On the other hand, the obtained HOMO-LUMO gap in the case of molecule P was 2.30 eV, with the HOMO level more concentrated in the N-atom and O-atom, while its LUMO level covered the (*sp*^2^)-C-atoms from the naphthalene-moiety.

**Figure 11:**
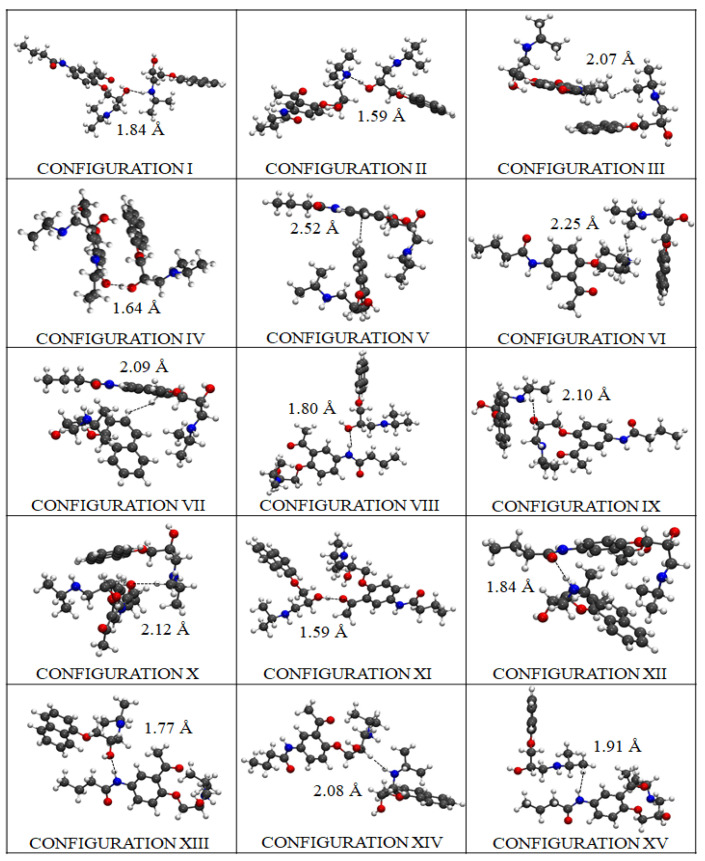
DFT results for the fifteen most stable thermodynamically drug–drug binding configurations (from I to XV) adopted by the beta-blockers A and P during the pharmacodynamic interactions (i.e., modeled in the absence of fibrinogen E-region). The beta-blocker atoms are depicted as ball and sticks and colored as follows: dark gray (C-atoms), red (O-atoms), blue (N-atoms), and light gray (H-atoms).

Then, we carried out DFT calculations for all the potential conformations adopted by the beta-blockers during the pharmacodynamic interactions, according to the ligand binding conformations and thermodynamic stability. To do so, the most stable drug–drug binding configurations of the beta-blockers were identified following several criteria for their prioritization based on the following DFT obtained results: drug–drug energy of binding (*E_b_*), drug–drug inter-atomic distances of binding (*d_A_*_–*P*_) for the molecules A and P, and the HOMO-LUMO energy difference gap (ΔHL) of each modeled system (see Table 1). In so doing, the fifteen most stable drug–drug binding configurations from a total of forty modeled configurations were found (see Figure 11).

**Figure 12:**
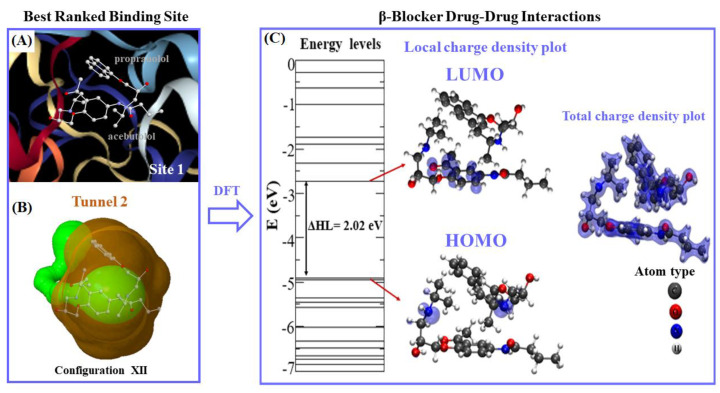
(**A**) Representation of the best-ranked drug–drug binding configuration XII formed by the A + P/fibrinogen complex in the fibrinogen E-region binding site 1. (**B**) Topological representation of the tunnel 2 (colored green) containing the best-ranked drug–drug binding configuration XII for molecules A + P interacting simultaneously at the same biophysical environment (ball and sticks representation) in binding site 1 (surrounding shaded orange region). (**C**) Energy levels and iso-surfaces of the local charge electronic density and total charge density plots obtained for the best-ranked drug–drug binding configuration XII (A + P). The electronic levels are depicted as solid lines with the HOMO-LUMO energy difference gap (ΔHL), and the density plots are shown, using orbital charge density iso-surfaces of 0.002 eV/Å^3^. The A and P atoms are depicted as ball and sticks and colored as follows: dark gray (C-atoms), red (O-atoms), blue (N-atoms), and light gray (H-atoms).

The binding energy |*E_b_*|-based distance is considered the most relevant DFT result to tackle the DDI between both beta-blockers. It is important to note here that the DDI phenomena strongly depend on the best thermodynamic stability of the configuration binding-poses adopted by the beta-blockers in the binding site 1. Regarding this, the DFT results point out that the best-ranked configuration binding-pose was achieved by configuration XII, which presented the highest drug–drug binding-energy value |*E_b_*| = 2.40 eV, with an HOMO-LUMO difference gap of ΔHL = 2.02 eV associated to an inter-atomic distance of interaction (***d***(O···H)***_A_*_–*P*_**) = 1.84 Å, established between the O**-**atom of molecule A and the H-atom of molecule P. Short inter-atomic distances such as O**···**H between molecules A and P in this configuration favor the non-covalent stabilization of the DDI system, forming a (O···H)-dipole induced-dipole interaction without involving the sharing of electrons. Details on this analysis are shown in Figure 12.

These DFT results go along with the previous docking results, since configuration XII is part of the clustered docking poses that exhibit higher frequency values for DDI in the fibrinogen E-region site 1 (i.e., at site 1 *k* occurrences = 100) as referred to before (see Figure 6D). Additionally, configuration XII exhibits a greater structural overlap between the aromatic moieties (i.e., benzene and naphthalene) compared with the remaining configurations (Figure 11). Interestingly, the second-best ranked drug–drug binding configuration corresponds to configuration IV, which present a slightly lower-energy value (|*E_b_*| = 2.36 eV) compared to the former one (XII), but in contrast, it shows a lower value for the inter-atomic distance O···H (***d*** (O···H)***_A–P_*** = 1.64 Å). Additionally, the slightly difference of 0.04 eV obtained for configuration IV in relation to the best drug–drug binding configuration XII (|*E_b_*| = 2.36 eV) may be explained by the presence of structural overlapping parallel geometry between the aromatic benzene ring of A and the naphthalene-moiety of P. This clearly indicates potential influence of the π–π stacking in the stabilization of the beta-blocker DDI in this case.

In this context, by analyzing the worst-ranked configuration XV with |*E_b_*| = 1.67 eV, one can observe a clear separation of the aforementioned aromatic-moieties, which are oriented in a different plane (please refer to Figure 11). Furthermore, the presence of β-blocker inter-atomic distance of interaction *d_A_*_−_*_P_*(H-H) = 1.91 Å and HOMO-LUMO difference ΔHL = 2.21 eV could negatively influence the stabilization of configuration XV, and thus decrease the DDI phenomena in the fibrinogen E-region site 1 compared to the best-ranked drug–drug binding configurations (XII > IV) (see Table 1).

Considering the electronic properties for the best-ranked configuration (XII) in the fibrinogen E-region (binding site 1), one can note locally concentrated charges in the LUMO of A mainly placed on the (*sp*^2^)-C-atoms of the aromatic benzene-ring and covering partially the O-atom that forms the oxo-moiety in the A molecule. This fact could significantly weaken the H-bond electron-acceptor properties of the oxo-moiety that interacts with the (H)-atom of the regulatory residue THR22: chain P:CA in the fibrinogen binding site 1. Therefore, in this way, it will affect the stabilization of the A-fibrinogen docking complex as well as significantly the pharmacological activity of the A when beta-blocker P interacts in the same biophysical environment (site 1). Regarding the HOMO, the local charge density of configuration XII is spread on the N-atoms simultaneously for both beta-blockers. However, the latter does not involve relevant interacting-atoms for stabilizing the A + P/fibrinogen complex in site 1. On the other hand, the results concerning the total charge density plots [52] showed that the charges were localized on the separated beta-blockers only without charge lines intercepting both molecules, thus confirming the absence of DDI under a covalent regime between the beta-blockers A and P. Additionally, the obtained DFT results for the binding energies (|*E_b_*|) for all the configurations of (A + P) were smaller than 2.50 eV indicating that the interactions occur without chemical bonds [58,59]. Furthermore, the total charge density plots show that the geometric structure and inter-atomic bond-distances are similar to the ones of the original isolated molecules, suggesting that the beta-blocker DDI in site 1 could be more intense mimicking traditional pharmacodynamic DDI phenomena.

### 2.5. Experimental Validation

Let us first discuss the critical aggregation concentrations (cac), which are defined as the concentration from which occurs the aggregate formation and strongly depends on the total concentration of the beta-blocker species present in the system (i.e., A and P). Herein, the cac values were obtained from breaks in plots of velocity vs. concentration of beta-blockers drug mixture in aqueous solutions. Variations of the cac with temperature for different molar ratios of the P/A mixtures were plotted (see Figure S6). Each plot appears to follow a U-shaped curve with a minimum at a certain temperature. Based on the Regular Solution Theory, it is possible to obtain *β*, the dimensionless intra-aggregate interaction parameter [62], which is related with the drug–drug interactions in the mixed aggregates and can be interpreted in terms of an energetic parameter that represents the excess Gibbs free energy of mixing: *β* = *N*_A_ (*W*_11_ + *W*_22_ − 2*W*_12_), in which *W*_11_ and *W*_22_ are the energies of interaction between molecules in the pure state and *W*_12_ is the interaction between the two beta-blockers. The value of *β* can be obtained from the experimental values plotted in Figure S6, by means of the Equations below.

(4) $$\frac{X_{1}^{2}\;ln\;[\alpha_{1}\;{\mathrm{cmc}}_{m}/(X_{1}\;{\mathrm{cmc}}_{1})]}{{\left(1-X_{1}\right)}^{2}ln\;[(1-\alpha_{1})\;{\mathrm{cmc}}_{m}/(1-X_{1})\;{\mathrm{cmc}}_{2}))]}=1$$

(5) $$\beta =\frac{ln\;[\alpha_{1}\;{\mathrm{cmc}}_{m}/(X_{1}\;{\mathrm{cmc}}_{1})]}{{\left(1-X_{1}\right)}^{2}}$$

Obviously, the *β* values depend on the temperature and mixing ratio of A plus P. For a mixing concentration ratio of 0.5 (1:1), which mimics the multicomponent docking complex formed by the two β-blockers ligands, the obtained values range from −0.41 to −0.89 kBT or equivalently −0.26 to −0.55 kcal/mol. Although the experimental values are quantitatively different to the theoretically obtained, from the qualitative point of view, this evidence fits with the phenomenological interpretation involved. In fact, negative *β* values indicate attractive synergic interactions, so both beta-blockers must attract each other in aqueous solutions [62,63].

On the other hand, density and ultrasound velocity measurements were included to obtain the adiabatic compressibility using the Laplace Equation:

(6) $$k_{S}=-\frac{1}{V}{\left(\frac{\partial V}{\partial P}\right)}_{S}=\frac{10^{-3}}{\rho \;u^{2}}$$

where *V*, *P*, and *S* refer to volume, pressure, and entropy, respectively. $k_{S}$ is the adiabatic compressibility coefficient, expressed in Pa^−1^ when the ultrasound velocity *u* is expressed in cm s^−1^ and the density in g cm^−3^ [64]. The isentropic apparent molal compressibility ($K_{\varphi }$) can be calculated from the following Equation:

(7) $$K_{\varphi }=-\frac{10^{3}(k_{S}-k_{S}^{0})}{m\;\rho_{0}}+k_{S}^{0}\;V_{\varphi }$$

The plots of $K_{\varphi }$ as a function of the total concentration and the drug–drug mixing ratio of A plus P can be found in Figure S7. Changes in the apparent molar compressibility of the aggregation processes were all negative, confirming the predominant role of the increase in hydrophobic hydration in the association of the monomers of the evaluated beta-blocker drugs [65,66]. The large increment in the compressibility is a consequence of vertical stacking of the molecules in the aggregate with the polar groups and the side chains arranged around the periphery of the stack. This type of association has been suggested for phenothiazine and other drugs based on NMR experimental studies.

## 3. Materials and Methods

### General Workflow

To begin with, the protein receptor file—i.e., fibrinogen E-region—was withdrawn from the Protein Data Bank [67], PDB ID: 1JY2 with 1.4 Å of resolution and more than 98% similarity with the human fibrinogen E-region [26]. Then, the E-region PDB structure was prepared using the AutoDock Vina software [68], by including titration of the protonation states using the PROPKA 3.1 utility tool [69], followed by the addition of missing atoms with an overall optimization of the H-network employing its implemented PDB2PQR 2 algorithm. Next, the ligands (beta-blocker drugs) were obtained from the PubChem Substance and Compound databases [70]—i.e., A: hydrochloride molecule (PubChem CID:1978; MF: C_18_H_28_N_2_O_24_) and P: hydrochloride molecule (PubChem CID:62882; MF: C_16_H_22_CL_2_O_2_), and optimized using the semiempirical AM1 method implemented in MOPAC [71].

**Figure 13:**
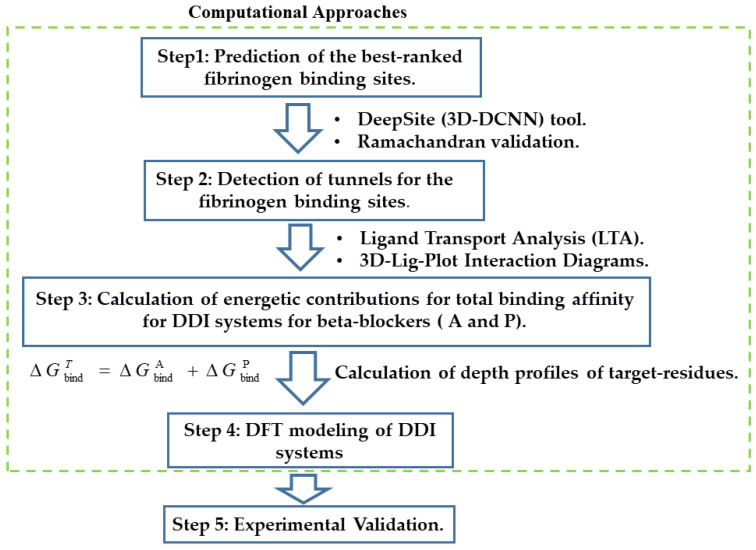
General workflow adopted in this study.

The following fifth-step procedure was adopted for addressing the study of the drug interactions between the beta-blockers A and P with the fibrinogen E-region binding-sites (see Figure 13).

**Step 1: Prediction of the binding sites for fibrinogen.** This proceeds as follows.

(i) Predict the binding sites for the fibrinogen E-region by applying an appropriate machine learning framework.
(ii) Select from the fibrinogen E-region binding sites found, the three best-ranked ones based on their highest druggability. For comparison purposes, ascertain as well the undruggable or “worst fibrinogen E-region binding site” to be used as reference control denoting absence of drug–drug interactions.
(iii) Validate the three best-ranked binding sites found by examining their Ramachandran plots.

**Step 2: Identification of tunnels for the fibrinogen binding sites**. Detect the tunnels or tiny cavities of the three best-ranked binding sites found, followed by a ligand transport analysis for the beta-blockers A and P on those, and inspection of 3D interaction diagrams for their target-residues.

**Step 3: Calculation of energetic contributions for binding affinity.** Ascertain the energetic contributions for the total binding affinity from beta-blocker DDI systems on the three best-ranked fibrinogen E-region binding sites and evaluate in addition their binding relevance before as well as after the beta-blocker DDI.

**Step 4: DFT modeling of beta-blocker drug–drug binding systems.** Evaluate the configurations of the best-ranked beta-blocker drug–drug binding systems, using DFT calculations.

**Step 5: Experimental validation.** Assess the reliability of the computational results by resorting to ultrasound density and velocity measurements.

**Step 1: Prediction of the binding sites for fibrinogen.** The task here is to predict the binding sites of the fibrinogen E-region, prior to the docking simulations. This was accomplished by applying the 3D DeepSite Convolutional Neural Networks (3D-DCNN) tool [32]. The latter considers all the crystallographic descriptors of a given binding site such as van der Waals surface of the fibrinogen cavities using a validated deep-learning neural network algorithm. Herein, the characterization and ranking of the predicted binding sites were established by considering the ability of these sites to simultaneously bind both beta-blocker drugs A and P at the same biophysical environment [18,19,20].

Then, the cartesian coordinates obtained from the volumetric maps of the fibrinogen binding sites were used to set up the docking box simulation for each fibrinogen E-region binding site. For site 1, the details of the box-simulation are: grid box-size with volume V = 559 Å^3^ (*X* = 64 Å, *Y* = 52 Å, *Z* = 52 Å) centered at *X* = 13.6 Å, *Y* = −1.10 Å, *Z* = 12.0 Å; whereas for site 2, these are: grid box-size with volume V = 475 Å^3^ (*X* = 24 Å, *Y* = 24 Å, *Z* = 24 Å) centered at *X* = 28.6 Å, *Y* = 2.80 Å, *Z* = 16.0 Å; and for site 3: grid box-size with volume V = 545 Å^3^ (*X* = 28 Å, *Y* = 28 Å, *Z* = 28 Å) centered at *X* = 0.2 Å, *Y*= −8.4 Å, *Z* = 5.7 Å. Finally, for the “worst fibrinogen binding site”, these are: grid box-size with volume V= 500 Å^3^ (*X* = 28 Å, *Y* = 28 Å, *Z* = 28 Å) centered at *X* = 38.6 Å, *Y* = 13.1 Å, *Z* = 31.1 Å. For this instance, an exhaustiveness docking parameter equal to 100 was used in all the cases [72].

To establish the relevance of each catalytic binding site, the druggability (D_g_) was estimated as the maximum energy variation of the fibrinogen E-region binding-sites affinities under beta-blocker drug interactions. D_g_ is defined as follows:

(8) $$D_{g}\approx -\Delta G_{\max}(\mathrm{Fibr}.\;\mathrm{site})\approx -\gamma \;(r)\;A_{\mathrm{Non}\text{-}\mathrm{polar}\;\mathrm{site}}\times \frac{A_{\mathrm{site}\text{-}\mathrm{druglike}}}{S_{\max\;\mathrm{site}}}+C$$

where $\Delta G_{\max}(\mathrm{Fibr}.\;\mathrm{site})$ is similar to D_g_ [19], more commonly used in rational drug design to represent the maximum intrinsic affinity of the fibrinogen catalytic binding site, which fits to a linear-correlation between the binding-site surface area and the beta-blocker molecular weights under DDI conditions. Then, *γ* (=0.024 kcal/mol) stands for a constant related to solvent surface tension in the fibrinogen binding site, which depends on the surface curvature *r* (i.e., concave surface) of the fibrinogen binding site. The *A*_Non__-__polar site_ corresponds to an approximation of the beta-blockers desolvation components, whereas the *A*_site__-__druglike_ to the drug-like size (≈300 Å^2^). The *S*_max site_ depicts the normalized maximum solvent-accessible surface area of the fibrinogen site evaluated, and *C* is an empirical parameter that largely discriminates druggable from undruggable binding-sites when it is ca. zero [18,19,20].

**Validation of the fibrinogen binding sites**. To validate the 3D *X*-ray crystallographic structure of the predicted best-ranked fibrinogen E-region binding sites —namely: sites 1, 2, and 3— a Ramachandran diagram analysis Psi (ψ) vs. Phi (φ) was carried out [38]. Here, it should be noted that Ramachandran diagrams may prevent obtaining false positives from docking complexes by confirming the absence of restricted-flexibility in the target-residues making up the fibrinogen E-region binding sites.

**Step 2: Identification of tunnels for the fibrinogen binding sites.** Detection of tunnels for each fibrinogen E-region binding site was performed using the Caver 1.0 software [36]. One of the most relevant tasks here is to appropriately select the tunnel starting point residues, which are typically placed within the active site. For this purpose, we considered the three fibrinogen binding sites with the highest D_g_ values, and redundant tunnels were removed in order to set the starting point residues, namely: at site 1: N:48 N:49 N:50 N:51 P:19 P:20 P:21 P:22 O:81 O:83 O:84 O:85 O:86 Q:48 Q:49 Q:50 Q:51 S:19 S:20 S:21 S:22 R:81 R:83 R:84 R:85 R:86; at site 2: O:76 O:78 O:79 Q:54 Q:57 Q:60 Q:61 S:26 S:29 S:30 S:33 S:34; and at site 3: N:54 N:57 N:60 N:61 P:26 P:29 P:30 O:64 P:33 R:76 R:78 R:79. The algorithm implemented in this software allows us to calculate the geometry of a given binding site and the target-residues composing the tunnel, as well as searching the tunnel pathways with the minimal radius and the lowest cost based on a Voronoi tessellation procedure [38]. In all the cases, the particular detection parameters applied here are minimum probe radius = 0.9 Å, shell depth = 4 Å, shell radius = 3 Å, clustering threshold = 3.5 Å, maximal distance = 3 Å, and desired radius = 5 Å.

**Beta-blocker ligand-transport analysis.** Afterwards, we carried out a ligand transport analysis (LTA) on the previously detected tunnels using the CaverDock software [54]. The LTA algorithm is able to generate the trajectories from the individual free binding energy (Δ*G*_bind_) by performing an iterative docking of the beta-blockers (A or P) to every slice of the tunnel. In so doing, we employed the partially restrained AutoDock Vina scoring function [65] that considers two relevant parameters—i.e., (i) the discretization delta that defines the distance between two slices centers of the tunnel, and (ii) the calculation mode that defines which ligand restraints will be set as rotation restriction coupled with backtracking to ensure a forward continuous movement. For that, the beta-blocker free binding energy trajectories were set as default parameters, which is strongly recommended in pharmacodynamic DDI studies [54,68].

In addition, to evaluate the efficiency of the simultaneous transport of the beta-blockers A and P through the different tunnels, the biophysical throughput parameter (***T***) was estimated according to Equation (9). The throughput of a given pathway can adopt the form of the equation below when the ligand transport trajectory has continuous variation through the tunnel radius [54].

(9) $$T=e^{\int_{0}^{L}\;r{\left(l\right)}^{-n}dl}$$

In this Equation, *L* represents the total path length with radius *r*, the function *r*(*l*) defines the largest radius for absence of collisions or steric clashes along the beta-blocker drug–drug trajectories, and *n* ∈ *N*, though its default value is *n* = 2 for tunnels with relatively large width. Here, the “cost” is obtained by numerically integrating the exponent using the trapezoidal rule with a uniform grid, a minimum number of trapezoids (=8), and a minimum grid distance (=0.1 Å). As such, the ***T*** values range from 0 to 1 [54].

**Analysis of 3D interaction diagrams.** To evaluate the relevant beta-blocker DDI between A plus P with the fibrinogen E-region, 3D interaction diagrams were plotted and analyzed using the ezCADD software [44,45]. This software determines the non-covalent intermolecular interactions in each receptor-ligand complex. Then, its utility tool named ezLigPlot automatically represents schematic 3D-interaction diagrams for the van der Waals hydrophobic, H-bond, cation-π, and π–π stacking interactions if any, along with the corresponding interatomic distances for all the docking complexes [44,45].

**Step 3: Calculation of energetic contributions for binding affinity.** The total binding affinity for the beta-blockers drug–drug/fibrinogen E-region complexes was computed as the sum of the individual binding energies of the beta-blockers A and P ($\Delta G_{\mathrm{bind}}^{A}$ and $\Delta G_{\mathrm{bind}}^{P}$) in the three best-ranked fibrinogen E-region binding sites (from now on, denoted as sites 1, 2, and 3). To this end, the empirical force-field parameters of the Autodock Vina scoring function were used to ascertain the effect of the different thermodynamic contributions on the total binding affinity [41,42,49,55,56]. That is, the individual binding energies were obtained by summing up the different contributions, as shown below for e.g., the beta-blocker acebutolol (A)—Equation (10), and then added together to gather the total binding affinity ($\Delta G_{\mathrm{bind}}^{T}$; Equation (11)).

(10) $$\Delta G_{\mathrm{bind}}^{A}=\Delta G_{\mathrm{Gauss}1}^{A}+\Delta G_{\mathrm{Gauss}2}^{A}+\Delta G_{\mathrm{repulsion}}^{A}+\Delta G_{H\text{-}\mathrm{bond}}^{A}+\Delta G_{\mathrm{hydrophobic}}^{A}+\Delta G_{\mathrm{rot}}^{A}$$

(11) $$\Delta G_{\mathrm{bind}}^{T}=\Delta G_{\mathrm{bind}}^{A}+\Delta G_{\mathrm{bind}}^{P}$$

Notice that in Equation (10), the steric interaction-based Vina scoring function is calculated using three terms—i.e., two attractive Gaussian functions distance dependent terms (Δ*G*_Gauss1_ and Δ*G*_Gauss2_), and one repulsive parabolic interaction term (Δ*G*_repulsion_)—which reproduce the canonical Lennard–Jones interaction shape of beta-blockers with fibrinogen [41,42,49,55,56,73]. The Δ*G*_H-bond_ term accounts for the pair of donor and acceptor hydrogen-bonds. The fourth term Δ*G*_hydrophobic_ aims at evaluating the non-covalent van der Waals interactions [33,34,35]. Lastly, the Δ*G*_rot_ term holds for the number of rotatable bonds/torsions in the beta-blocker ligands.

The total binding affinity is, therefore, used to quantify the DDI when both beta-blockers interact simultaneously at the same biophysical environment and crystallographic plane of the three best-ranked fibrinogen E-region binding sites. When $\Delta G_{\mathrm{bind}}^{T}$ > 0 kcal/mol, it is classified as energetically unfavorable, pointing either to extremely low or complete absence of affinity of the formed complexes, otherwise it is categorized as medium to high binding affinity. Additional details about the thermodynamic force field parameters of the Autodock Vina scoring function used in this work can be seen in [33,34,35]. An additional control simulation experiment was carried out to evaluate the maximum per atom-energy contribution to the individual binding affinity of the A and P separately in the three best-ranked fibrinogen E-region binding sites.

**Depth profiles of fibrinogen target-residues.** In order to evaluate the binding relevance of the beta-blocker DDI, we performed a depth profile analysis of the target-residues (i) including the target-chains (***c***) making the tunnels [37,39,40,74]. To this end, the residue depth is represented by the *D*_[*c*,__*i*]_ parameter, defined according to the general following Equation in compact notation:

(12) $$D_{\left[c,i\right]}=\prod_{c}\left[1-\prod_{c}\left(1-q_{i}^{c}\right)\right]\times S_{\max}$$

where $q_{i}^{c}$, known as the depth quasi-sequence-order descriptor, represents an adjusted binding probability-based weighted average derived from the distance matrix between the twenty standard amino-acids, and displays a linear correlation with the maximum solvent accessibility *S*_max_—i.e., the percentage of predicting cavity waters to be displaced from the cited critical residues ***i*** in the main-chains ***c*** [37,39,40,74]. Additionally, the $q_{i}^{c}$ descriptor is a function of the quasi-sequence-position-order descriptor (*J_i_*), so-called conservation score, which is related to the position and occurrence of the residue ***i*** in the sequence of the chain. More details about these descriptors are discussed in the former section.

It is also important to highlight that the *D*_[*c*,_ *_i_*_]_ parameters for two different chains are independent from each other. Our hypothesis here is that the depth profile of target-residues (i) surrounding tunnels can significantly modulate the beta-blocker drug–drug interactions. Following this idea, in the present work, three categories were defined to evaluate the binding relevance of the fibrinogen target-chains, taking into account the number of chains (***c***) composing the tunnels involved in the beta-blocker DDI, namely: ***c*** = 6 (highest relevance), ***c*** = 3–4 (medium relevance), and ***c*** = 1–2 (low relevance).

**Step 4: DFT modeling of beta-blocker drug–drug binding systems.** A DFT study of the configurations for the best-ranked beta-blocker drug–drug binding complexes was performed [75]. To this end, the Kohn–Sham Equations were solved with numerical atomic orbitals using the SIESTA code [57,76]. Specifically, the Kohn–Sham orbitals are expanded in a finite basis set of numerical pseudoatomic orbitals, the latter being described by double-zeta polarization (DZP) functions [77]. Correlation energies were set by the local density approximation (LDA), as proposed by Perdew and Zunger [77]. In fact, the LDA approach has been shown to be more suitable than the generalized gradient approximation (GGA) to study weakly interacting systems with the presence of π-π stacking interactions [78].

Improved Troullier–Martins pseudopotentials were used to describe the interactions between the core and the valence electrons [58,59,61], and for the molecular orbitals a localized DZP polarization basis set was implemented. For all the beta-blocker DDI systems, a cutoff value of 200 Ry was used to represent the charges density in the grid integration. The optimization of the systems’ structure was performed by a conjugate gradient method, the beta-blocker atomic positions being fully relaxed until the remaining forces acting on them dropped below 0.05 eV/Å.

The DFT-binding energies ($E_{b}$) between the beta-blockers A and P were calculated according to the following Equation [58,59,61]:

(13) $$E_{b}=-(E\;{[A+P]}_{\mathrm{total}}-E\;\left[A\right]-E\;\left[P]\right)$$

where $E\;{[A+P]}_{\mathrm{total}}$ stands for the total DFT energy of the beta-blocker drug–drug system, and E [A] or E [P] for that of the isolated beta-blocker molecules A or P, respectively.

**Step 5: Experimental validation.** To validate our theoretical results on beta-blocker DDI, the ultrasound velocities and densities were continuously, simultaneously, and automatically measured by using a DSA 5000 Anton Paar density and sound velocity analyzer. This method allows the determination of the concentration of two or more component solutions using the most accurate results of density for interacting components such as beta-blocker DDI at the same time and experimental conditions. This equipment possesses a vibrating tube for density measurements and a stainless-steel cell connected to a sound velocity analyzer with a resolution of ±10^−6^ g cm^−3^ and 10^−2^ m s^−1^, respectively. Both the speed of sound and density are extremely sensitive to the temperature, so this was kept constant within ±10^−3^ K through a Peltier system. The reproducibility of the densities and ultrasounds measurements is ±10^−6^ g cm^−3^ and 10^−2^ ms^−1^, respectively.

## 4. Conclusions

In this paper, we presented for the first time a combination of molecular docking and DFT results of the pharmacodynamic DDI between two beta-blockers (A and P) on the relevant catalytic binding-sites of the fibrinogen E-region. Those results showed that the three most relevant fibrinogen E-region binding sites exhibit from high to low druggability in the order: site 1 (D_g_ = 0.81) > site 2 (D_g_ = 0.54) > site 3 (D_g_ = 0.39), as well as complying with the mutual overlap criterion for being the best-ranked binding-sites, in contrast with the “worst fibrinogen binding site” with D_g_ = 0.08. Furthermore, the crystallographic validation based on Ramachandran diagrams shows that 100% of the catalytic binding residues associated with the transport of both beta-blockers were correctly categorized as conformationally favored residues with total absence of residues with restricted flexibility, thus confirming to be not false positives docking results. Furthermore, the binding free energy trajectories and total affinity obtained for the best-ranked beta-blocker drug–drug docking complexes revealed a spontaneous thermodynamic stabilization following the site-affinity order: site 1 > site 2 > site 3. The most dominant energy contributions to the total affinity of the DDI complexes (beta-blocker drug–drug/fibrinogen E-region) were provided by the pair of steric distance-dependent interactions (Gauss2 > Gauss1). In addition, the 3D-lig-plot interaction diagrams for the beta-blocker DDIs showed that the complex drug–drug stabilization was mostly influenced by the contributions of a high number of hydrophobic atoms (acebutolol > propranolol) in the fibrinogen site 1 (tunnel 2). Additionally, the later site was identified as the most preferred biophysical environment for beta-blocker DDIs involving all chains (N, O, P, Q, R, S) of the fibrinogen E-region structure and target residues with high depth-values. Along with these findings, the DFT results demonstrated that the best-ranked drug–drug configuration binding-pose (configuration XII) in the fibrinogen E-region binding site 1 does not involve the formation of covalent bonds between both beta-blockers. In addition to this, the experimental results supported the occurrence of hydrophobic DDIs between A and P. Indeed, the obtained values for the intra-aggregate parameter corroborate the docking results, and the adiabatic compressibilities match well with the DFT results suggesting that the coupling between aromatic moieties of both beta-blockers is key during the interaction process. Here, it should be stressed that in spite of DFT calculations based on the LDA approach being able to provide computationally efficient access to many ground-state properties of fairly large-size systems, in particular of those involving π–π interactions, their inability to accurately describe nonlocal dispersion forces is well known. Therefore, it is likely that a dispersion-corrected DFT approach will be needed for accurately describing the non-covalent interactions of the present beta-blockers. Efforts to address such limitation are currently underway in our research group.

In sum, the present work opens new perspectives in computational polypharmacology modelling of DDI, which can help to avoid the toxicological effects associated to the concomitant administration of beta-blockers. Moreover, the relevant mechanistic information gathered shall speed up decision making in rational drug-design.

## Figures and Tables

**Table 1:** Energy of binding (*E_b_*), lowest β-blocker inter-atomic distance of interaction (*d_A__–P_*), and HOMO-LUMO gap (ΔHL) obtained for the fifteen most stable configurations of beta-blocker DDI systems ordered according to their pharmacodynamic relevance (DFT results obtained by modeling DDI in the absence of fibrinogen E-region molecule).

Ranking	Configuration	|*E_b_*| (eV)	ΔHL (eV)	*d_A__–P_*(Å)
1	XII	2.40	2.02	1.84 (O-H)
2	IV	2.36	2.11	1.64 (O-H)
3	II	1.99	1.87	1.59 (N-H)
4	XIII	1.99	2.26	1.77 (H-O)
5	XIV	1.99	2.15	2.08 (N-H)
6	XI	1.97	1.63	1.59 (O-H)
7	VI	1.92	1.90	2.25 (N-H)
8	VII	1.88	2.13	2.09 (H-H)
9	X	1.84	2.29	2.12 (O-H)
10	VIII	1.83	2.30	1.80 (H-O)
11	III	1.82	2.22	2.07 (H-H)
12	I	1.76	1.47	1.84 (O-H)
13	IX	1.71	2.20	2.10 (O-H)
14	V	1.67	1.97	2.52 (C-H)
15	XV	1.67	2.21	1.91 (H-H)

## Supplementary Materials

The following are available online, Figure S1: Prediction of the worst catalytic binding site of fibrinogen E-region; Figure S2: Cartoon representation of beta-blocker interactions in the worst catalytic binding site of fibrinogen E-region; Figure S3: Graphical breakdown of the different binding energy contributions of beta-blocker interactions in the worst catalytic binding site of fibrinogen E-region; Figure S4: Representation of the per atom energy contributions of beta-blocker interactions in the worst catalytic binding site of fibrinogen E-region; Figure S5: Druggability-depth-maximum solvent accessibility relationship of the critical target-residues of fibrinogen E-region binding sites (sites 1, 2, and 3), Figure S6: Critical aggregation concentrations; Figure S7: Graphical representation of the apparent molal compressibility (K*_φ_*) vs. total concentration; Figure S8: Representation of the van der Waals surface for the predicted fibrinogen E-region binding sites with the corresponding tunnels.
