# Supplementary material for: Targeting Beta-Blocker Drug–Drug Interactions with Fibrinogen Blood Plasma Protein: A Computational and Experimental Study
Source: Molecules. 2020 Nov 19;25(22):5425. doi: 10.3390/molecules25225425 (PMC7699576; doi:10.3390/molecules25225425)
Supplement: Supplementary file 1 [file molecules-25-05425-s001.pdf]

Supplementary Material

Targeting beta-blocker drug-drug interactions with fibrinogen blood plasma protein: A computational and experimental study

Michael González-Durruthy [1,2,*], Riccardo Concu [1], Laura F. Osmari Vendrame [3], Ivana Zanella [3], Juan M. Ruso [2] and M. Natália D.S. Cordeiro [1,*]

[1] LAQV-REQUIMTE, Department of Chemistry and Biochemistry, Faculty of Sciences, University of Porto, 4169-007 Porto, Portugal; riccardo.concu@fc.up.pt
[2] Soft Matter and Molecular Biophysics Group, Department of Applied Physics, University of Santiago de Compostela, 15782 Santiago de Compostela, Spain; juanm.ruso@usc.es
[3] Post-Graduate Program in Nanoscience, Franciscana University (UFN), 97010-032 Santa Maria, RS, Brazil; laura.o.vendrame@gmail.com (L.F.O.V.); ivanazanella@gmail.com (I.Z.)
* Correspondence: michael.durruthy@fc.up.pt (M.G.-D.); ncordeir@fc.up.pt (M.N.D.S.C.); Tel.: +351-220402502 (M.N.D.S.C.)

Academic Editor: Micholas Dean Smith
Received: 16 October 2020; Accepted: 17 November 2020; Published: 19 November 2020

Table of contents

Figure S1. Prediction of the worst catalytic binding site of fibrinogen E-region.

Figure S2. Cartoon representation of beta-blocker interactions in the worst catalytic binding site of fibrinogen E-region.

Figure S3. Graphical breakdown of the different binding energy contributions of beta-blocker interactions in the worst catalytic binding site of fibrinogen E-region.

Figure S4. Representation of the *per* atom energy contributions of beta-blocker interactions in the worst catalytic binding site of fibrinogen E-region.

Figure S5. Druggability-depth-maximum solvent accessibility relationship of the critical target-residues of fibrinogen E-region binding sites (sites 1, 2, and 3).

Figure S6. Critical aggregation concentrations.

Figure S7. Graphical representation of the apparent molal compressibility (K_ϕ) *vs*. total concentration.

Figure S8. van der Waals surface representation of the predicted fibrinogen E-region binding sites with the corresponding tunnels.

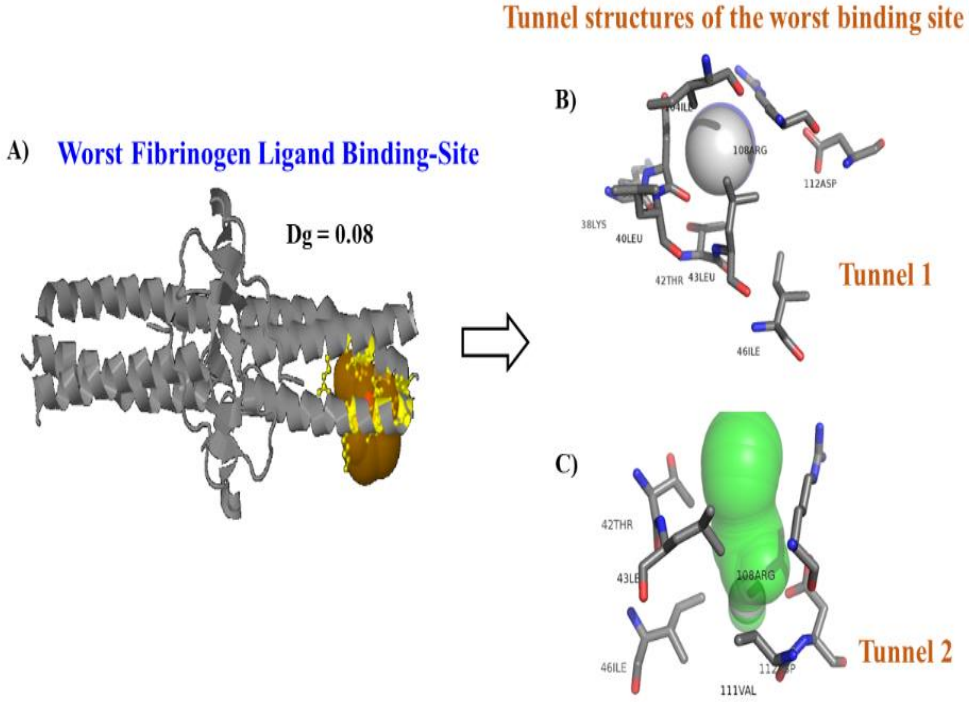

Figure S1. (**A**) Prediction of the worst catalytic binding site of fibrinogen E-region based on the druggability degree (Dg = 0.08). (**B**) and (**C**) Tunnel structures of the worst binding site of fibrinogen E-region, namely of tunnel 1 (gray) and of tunnel 2 (green) with the surrounding catalytic residues.

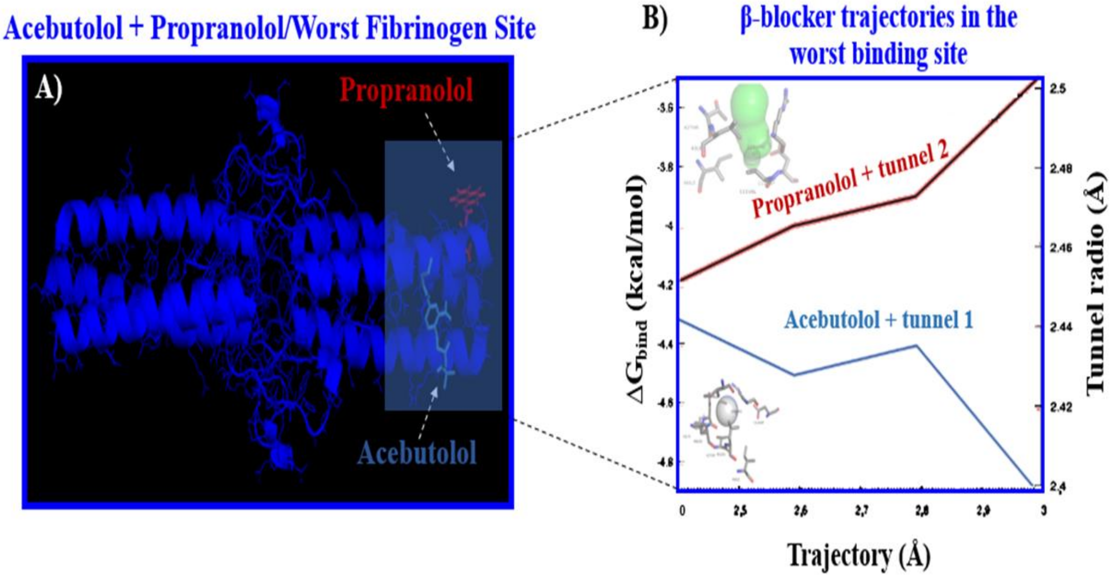

Figure S2. (**A**) Cartoon representation of beta-blocker interactions of acebutolol in tunnel 1 (blue) and propranolol in tunnel 2 (red) for the worst fibrinogen binding site (Dg = 0.08). (**B**) Binding profiles of beta-blocker trajectories showing the total absence of beta-blocker drug-drug interactions based on the non-interception between beta-blocker trajectories.

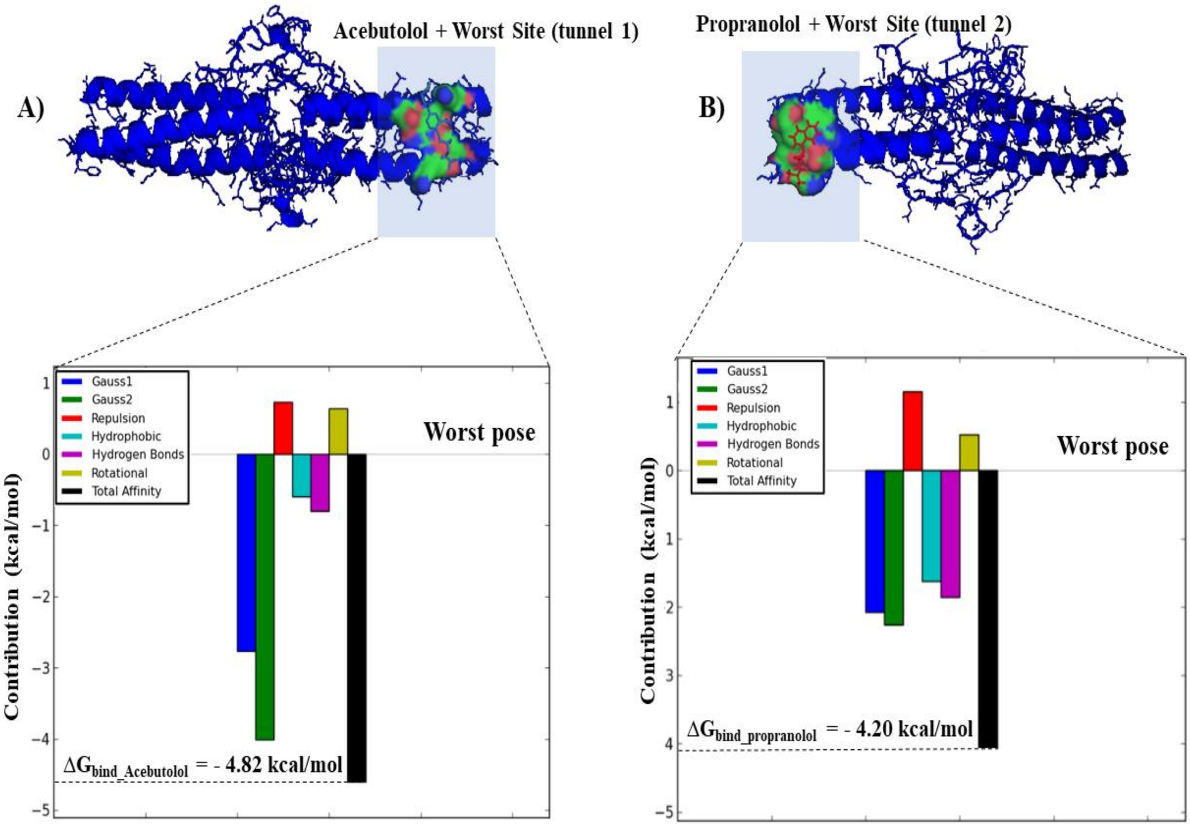

Figure S3. Graphical breakdown of the different binding energy contributions (ΔG_{Gauss1}, ΔG_{Gauss2}, $\Delta G_{repulsion}$, $\Delta G_{H\text{-}bond}$, $\Delta G_{hydrophobic}$, and $\Delta G_{rotational}$) to the individual binding affinity (ΔG_{bind}, kcal/mol) of the beta-blockers in the worst fibrinogen binding site (Dg = 0.08). (**A**) Acebutolol, (**B**) Propranolol.

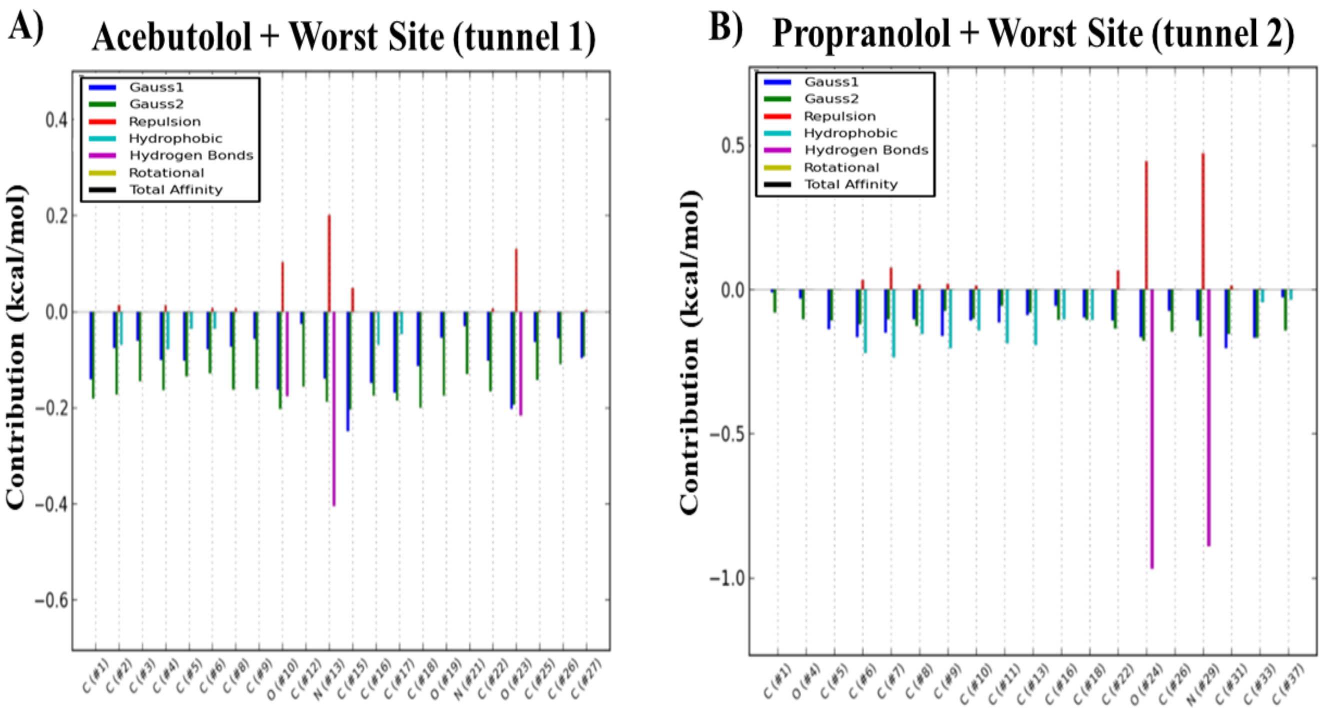

Figure S4. Representation of the *per* atom energy contributions (kcal/mol) to the individual binding affinity (ΔG_{bind}) of the beta-blockers in the worst fibrinogen binding site (Dg = 0.08). (**A**) Acebutolol, (**B**) Propranolol.

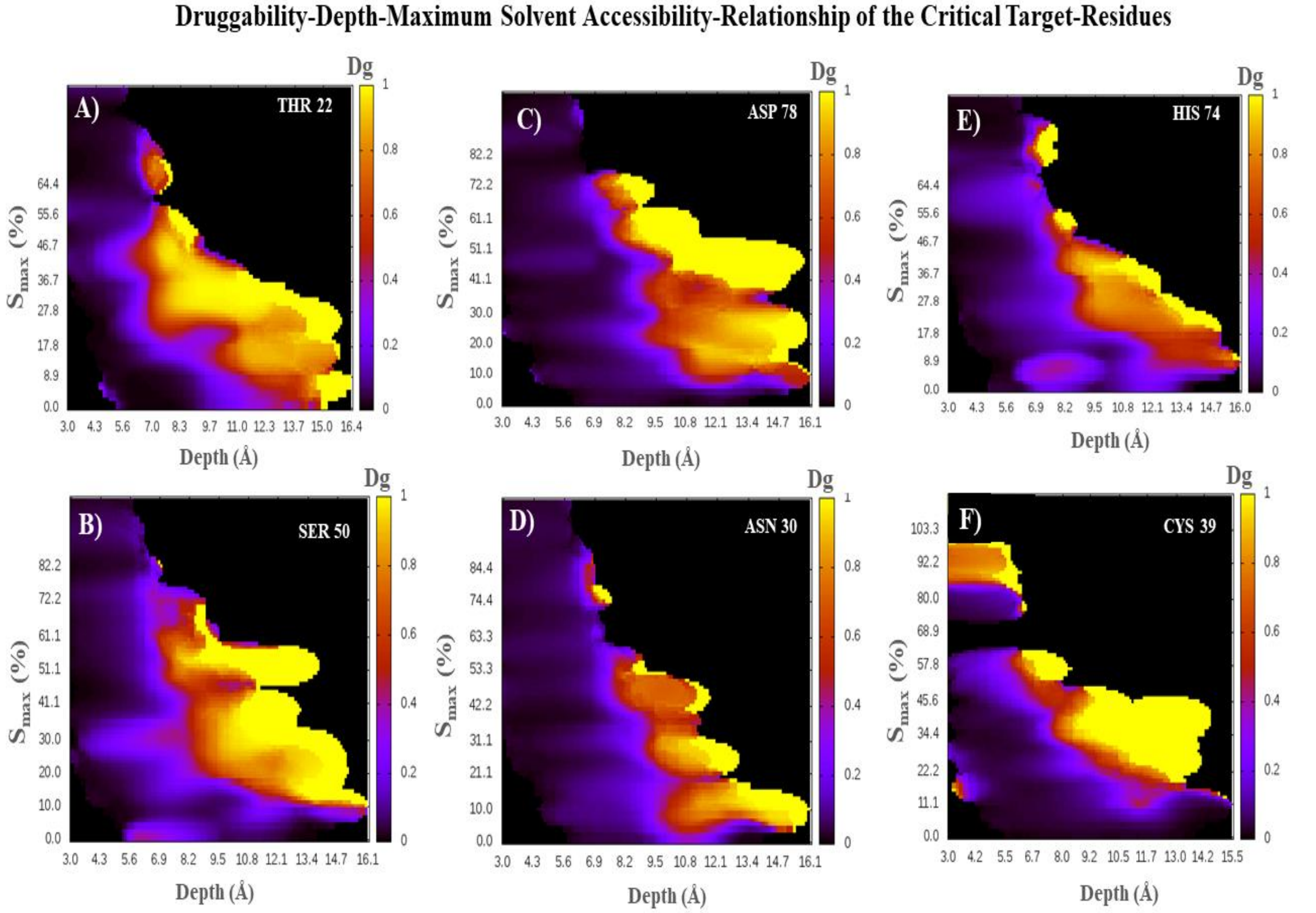

Figure S5. Druggability-depth-maximum solvent accessibility-relationship of the critical target-residues belonging to fibrinogen E-region binding sites (sites 1, 2, and 3). (**A**) TRH22, (**B**) SER50, (**C**) ASP78, (**D**) ASN30, (**E**) HIS74, (**F**) CYS39.

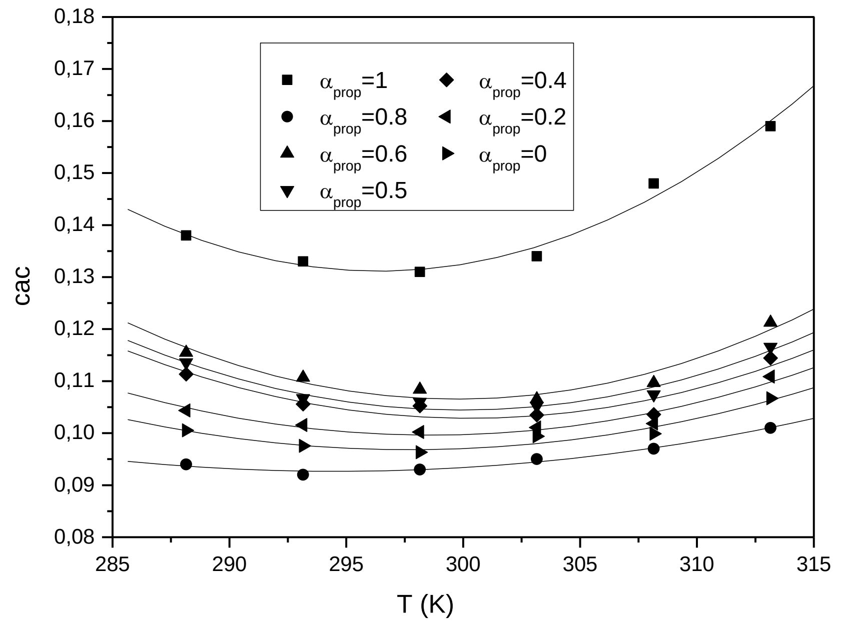

Figure S6. Critical aggregation concentrations (cac) as a function of propranolol concentration ratios (α_{prop}) obtained at different temperatures.

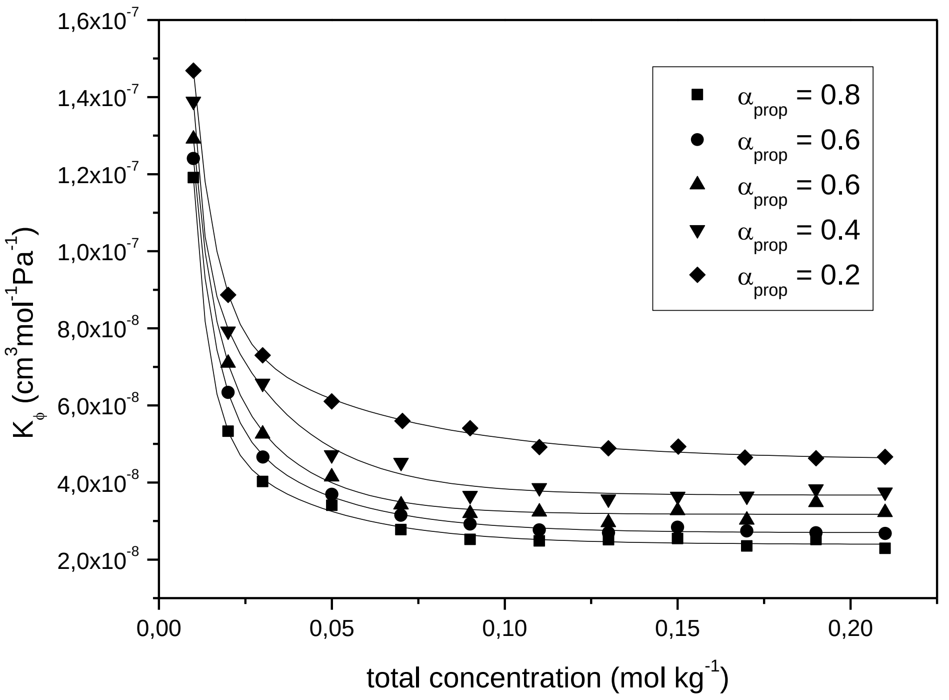

Figure S7. Graphical representation of the relationship between the apparent molal compressibility (K_ϕ) *vs*. total concentration at different propranolol concentration ratios (α_{prop}).

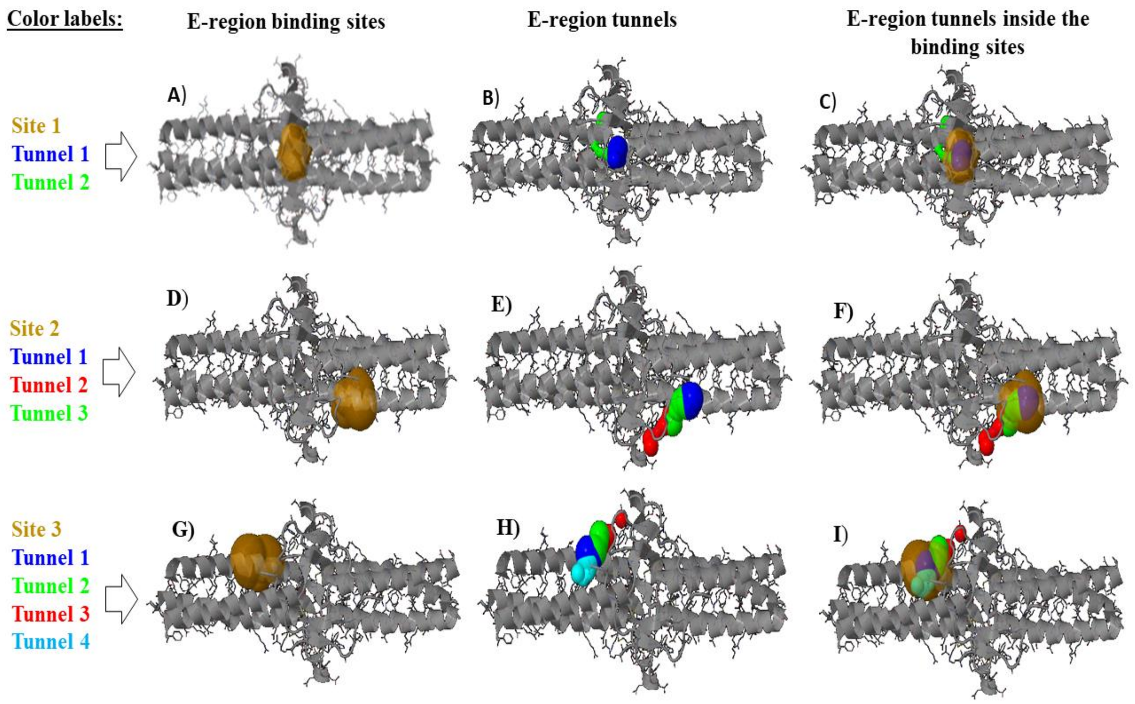

Figure S8. In the top, panels from (**A**) to (**C**) relate to the van der Waals surface representation of the predicted fibrinogen E-region binding site 1 with the corresponding tunnels (tunnels 1 and 2). In the middle, panels from (**D**) to (**F**) relate to the van der Waals surface representation of the predicted fibrinogen E-region binding site 2 with the corresponding tunnels (tunnels 1, 2 and 3). In the bottom, panels (**G**) to (**I**) relate to the van der Waals surface representation of the predicted fibrinogen E-region binding site 3 with the corresponding tunnels (tunnels 1, 2, 3 and 4). Binding sites are depicted as a transparent orange shadow region and the remaining colors correspond to the tunnels in all the cases.
